# A dual yet opposite growth-regulating function of miR-204 and its target XRN1 in prostate adenocarcinoma cells and neuroendocrine-like prostate cancer cells

**DOI:** 10.18632/oncotarget.3480

**Published:** 2015-03-08

**Authors:** Miao Ding, Biaoyang Lin, Tao Li, Yuanyuan Liu, Yuhua Li, Xiaoyu Zhou, Maohua Miao, Jinfa Gu, Hongjie Pan, Fen Yang, Tianqi Li, Xin Yuan Liu, Runsheng Li

**Affiliations:** ^1^ State Key Laboratory of Cell Biology, Institute of Biochemistry and Cell Biology, Chinese Academy of Sciences, Shanghai, China; ^2^ WHO Collaborating Center for Research in Human Reproduction, Shanghai, China; ^3^ Key Laboratory of Contraceptive Drugs and Devices of NPFPC, Shanghai Institute of Planned Parenthood Research, Shanghai, China; ^4^ Cancer Institute (Key Laboratory of Cancer Prevention and Intervention, China National Ministry of Education), The Second Afﬁliated Hospital, Zhejiang University School of Medicine, Hangzhou, Zhejiang, China; ^5^ Department of Urology, University of Washington, Seattle, WA, USA; ^6^ Department of Urology, Tongji Hospital, Tongji University School of Medicine, Shanghai, China; ^7^ The Obstetrics and Gynecology Hospital of Fudan University, Shanghai, China; ^8^ Institute of Reproduction and Development, Fudan University, Shanghai, China

**Keywords:** miR-204, XRN1, Prostate cancer

## Abstract

Androgen deprivation therapy in prostate cancer (PCa) causes neuroendocrine differentiation (NED) of prostatic adenocarcinomas (PAC) cells, leading to recurrence of PCa. Androgen-responsive genes involved in PCa progression including NED remain largely unknown. Here we demonstrated the importance of androgen receptor (AR)-microRNA-204 (miR-204)-XRN1 axis in PCa cell lines and the rat ventral prostate. Androgens downregulate miR-204, resulting in induction of XRN1 (5′-3′ exoribonuclease 1), which we identified as a miR-204 target. miR-204 acts as a tumor suppressor in two PAC cell lines (LNCaP and 22Rv1) and as an oncomiR in two neuroendocrine-like prostate cancer (NEPC) cell lines (PC-3 and CL1). Importantly, overexpression of miR-204 and knockdown of XRN1 inhibited AR expression in PCa cells. Repression of miR-34a, a known AR-targeting miRNA, contributes AR expression by XRN1. Thus we revealed the AR-miR-204-XRN1-miR-34a positive feedback loop and a dual function of miR-204/XRN1 axis in prostate cancer.

## INTRODUCTION

PCa is the most common malignancy affecting males in western countries, and it is the second leading cause of cancer deaths worldwide [1]. Although androgen deprivation treatment (ADT) has been proven effectively to suppress the tumor growth and progression of androgen-sensitive PCa, most of those androgen-sensitive PCa will eventually develop the resistance to ADT and become castration-resistant prostate cancer (CRPC), in which up-regulation of androgen signaling pathway is believed to play an important role [2-4].

Most of PCa is characterized as prostatic adenocarcinoma (PAC) with luminal cell features and expression of AR and prostate-specific antigen (PSA) [5]. Interestingly, PAC usually contains a small population (usually ~1%) of scattered neuroendocrine-like prostate cancer (NEPC) cells that do not express AR and PSA [6]. Furthermore, as a subtype of NEPC cells, small cell neuroendocrine carcinoma (SCNC) is often seen in patients with advanced disease, and is composed of pure neuroendocrine (NE) tumor cells [7] that express PCa stem cell marker CD44 [8-10]. Importantly, studies have shown that ADT may contribute to development of CRPC [11-13], in which the focal NED within the tumors raises and levels of NE-derived peptides such as neuron-specific enolase (NSE) and chromogranin-A (CgA) in the serum of CRPC patients are induced [14]. Consistent with this, studies indicated that suppression of AR expression is required for NED of cultured PAC cells [15, 16]. It is believed that cancerous NE cells secrete a variety of growth factors that can promote the proliferation of adjacent PAC cells via a paracrine mechanism in an androgen-ablated environment [17, 18], accounting for androgen-independent growth of PCa. However, the mechanism by which NED is induced after ADT still remains largely unclear.

Pathogenic, diagnostic and prognostic roles of miRNAs have been reported before in PCa. Studies showed that some miRs can act either as oncomiRs or oncosuppressors, and their expression can be regulated by androgen in PAC cells [19-22]. However, regulatory roles of miRs in NEPC cells are poorly understood. In the present study, we identified an AR-miR-204-XRN1 signaling axis in PCa cells, and revealed its dual yet opposite role in mediating growth of PAC and NEPC cells.

## RESULTS

### miR-204 expression is down-regulated by Androgen in both PAC and NEPC Cells

**Figure 1:**
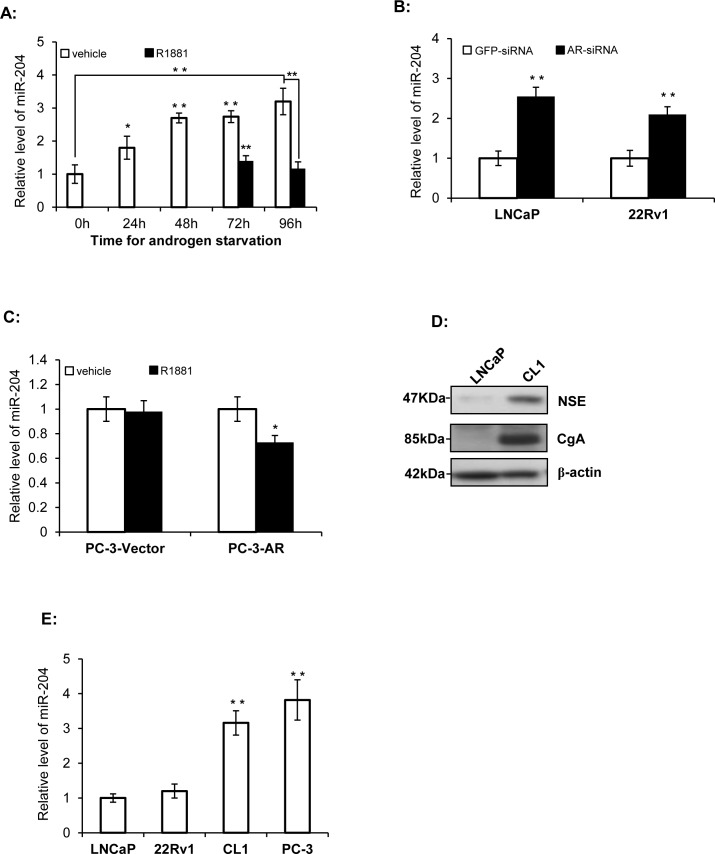
miR-204 expression is down-regulated by AR signaling A. Relative miR-204 levels as measured by RT-qPCR in LNCaP cells in the presence and absence R1881 (1.0 nM). B. Silencing of AR up-regulates miR-204 expression in LNCaP cells and 22Rv1 cells. Shown are RT-PCR results. C. The effect of exogenously-expressed AR on miR-204 expression in PC-3 cells. Shown are miR-204 RT-PCR results in PC-3 cells transfected with AR (PC-3-AR) and control vector (PC-3-vector). D. Immunoblotting analysis of expression of NSE and CgA in LNCaP cells and CL1 cells. E. Relative levels of miR-204 in untreated PCa cell lines as indicated. The levels of miR-204 were normalized to the levels measured in LNCaP cells. Bar, mean±SEM; * p<0.05, **p<0.01, n=3.

To study the possible impact of androgen on the miR's expression in PAC cells, we first used a miRNA array to compare miR expression of LNCaP cells in the presence and absence of androgen treatment. miR-204 was one of several miRs that was down-regulated by androgen (Supplementary Table 2). Consistent with this, our results showed that miR-204 levels increased gradually after the LNCaP cells were incubated in the medium with charcoal-stripped FBS that was depleted of androgen (Fig. 1A). Subsequently, after the synthetic androgen analog R1881was added to the culture at 48 and 72 hours post androgen withdrawal, the level of miR-204 expression decreased when compared to the control (Fig. 1A). Furthermore, when an AR-siRNA was transfected into 22Rv1 (an androgen-independent but androgen responsive PAC cell line [30]) and LNCaP cells, miR-204 expression increased by 2.51 folds and 2.12 folds, respectively, compared to those cells transfected with control GFP-siRNA (Fig. 1B). Together, these results indicate that androgen down-regulates miR-204 in PAC cells.

PC-3 cell line represents a NEPC cell line without endogenous AR expression [8]. We previously reported that PC-3 cells with a forcedly-expressed AR have an AR-regulated gene expression profile that is different from that in LNCaP cells [24]. As shown in Fig. 1C, miR-204 expression was also suppressed by R1881 in PC-3 cells transfected with AR, but not in PC-3 cells transfected with the control vector, indicating that inhibition of miR-204 expression in these cells is AR-dependent.

We next studied expression of miR-204 in NEPC cells, which do not expressed AR, by comparing their miR-204 expression with that in PAC cells. PC-3 and CL1 cells were used in this approach. PC-3 cell line represents a cell line of SCNC [8], while CL1 was derived from LNCaP cells through long-term *in-vitro* androgen-deprivation [23], in which LNCaP cells undergo NED [31]. Therefore CL1 cells might be a NEPC cell line, due to its high expression of CD44 [32], a feature of PCa cells with NE phenotype [8-10]. In addition, we previously performed a comprehensive expression profiling analysis of LNCaP and CL1 cells using the massive parallel signature sequencing (MPSS) technology [33], and identified 2088 MPSS signatures that are differentially expressed significantly (P<0.001) as listed in the Supplementary Table 2 of the publication [33]. Recently, Beltran et al. identified 1035 genes that are differentially expressed between NEPC and PCa tissues using RNA-seq [34]. Based on above information, we further compared the differentially expressed list of CL1 and LNCaP with the differentially expressed list of NEPC and CaP tissues, and identified an overlap of 35 genes. Among these 35 genes, 28 (80%, 28/35) of them changed in the same direction in the comparison (Supplementary Table 3), suggesting that CL1 and NEPC are more similar to each other than LNCaP and NEPC. Moreover, we detected a much higher expression of NSE and CgA, the two NE markers, in CL1 cells compared with that in LNCaP cells (Fig. 1D). All these support that CL1 represents a NEPC subclone of LNCaP cells.

Finally, our results further showed that miR-204 expression was significantly higher in the two NEPC cell lines (i.e. PC-3 and CL1) than that in the two PAC cell lines (i.e. LNCaP and 22Rv1) (Fig. 1E). Taken together, our results strongly suggest that AR is the key suppressor of miR-204 expression in PCa cells.

### miR-204 exhibits a dual regulation on PCa growth both *in vitro* and *in vivo*

**Figure 2:**
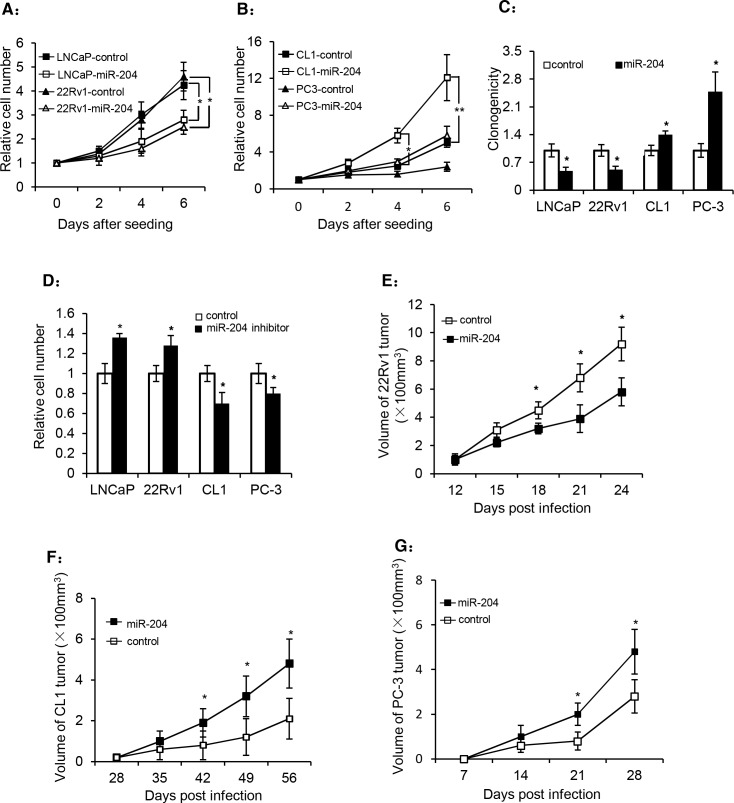
miR-204 has a dual regulation on the growth and colony formation of PCa cells (A and B) Cell growth of different PCa cell lines in the presence or absence of miR-204 overexpression by lentivirus. (C) Dual effect of miR-204 overexpression on the colony formation of PCa cells. (D) The effect of a miR-204 inhibitor on the growth of PCa cells. The four cell lines were transfected with the miR-204 inhibitor or a non-targeting control one day after they were seeded in 96-well plates (20 thousand cells each). The cells were used for cell counting 72 hours later. (E-G) The effect of miR-204 overexpression on prostate tumor growth rate in nude mice. 4 day after infected with miR-204-expressing virus or the control virus, 22Rv1 cells (E), CL-1 cells (F) and PC-3 cells (G) (2×10^6^ each) were injected subcutaneously into the right flank of male nude mice (8 mice/group). The tumor volume was measured at the indicated times (data are represented as the mean± SEM; * p<0.05; **p<0.01).

To investigate whether miR-204 affects the growth of PCa cells, we overexpressed miR-204 in different PCa cells, and our results showed that overexpression of miR-204 inhibited the growth of LNCaP and 22Rv1 cells (Fig. 2A), whereas it stimulated PC-3 and CL1 cell growth (Fig. 2B). Similarly, an opposite effect of miR-204 on the clonogenicity of LNCaP/22Rv1 and PC-3/CL1 cell lines was also observed (Fig. 2C). Moreover, when a miR-204 inhibitor was transfected into cells, it significantly stimulated the growth of LNCaP and 22Rv1 cells, but inhibited the growth of CL1 and PC-3 cells (Fig. 2D). Together, all these results demonstrated that miR-204 plays a dual yet opposite regulatory role in growth of different PCa cells *in vitro*.

To further evaluate the impact of miR-204 on PCa growth *in vivo*, we used a xenograft model in nude mice injected with the PCa cells infected with either miR-204-expressing virus or the control virus. Consistent with our *in vitro* observation (Fig. 2A-C), our results showed that overexpression of miR-204 in 22Rv1 tumor cells resulted in a time-dependent reduction of tumor volume (Fig. 2E). By contrast, overexpression of miR-204 significantly promoted the growth of CL1 (Fig. 2F) and PC-3 (Fig. 2G) tumors in nude mice. These results again demonstrated the dual yet opposite role of miR-204 in regulation of tumor growth of PAC and NEPC *in vivo*.

### Identification of XRN1, a potential target of miR-204 that participates in the dual regulation of PCa cell growth

**Figure 3:**
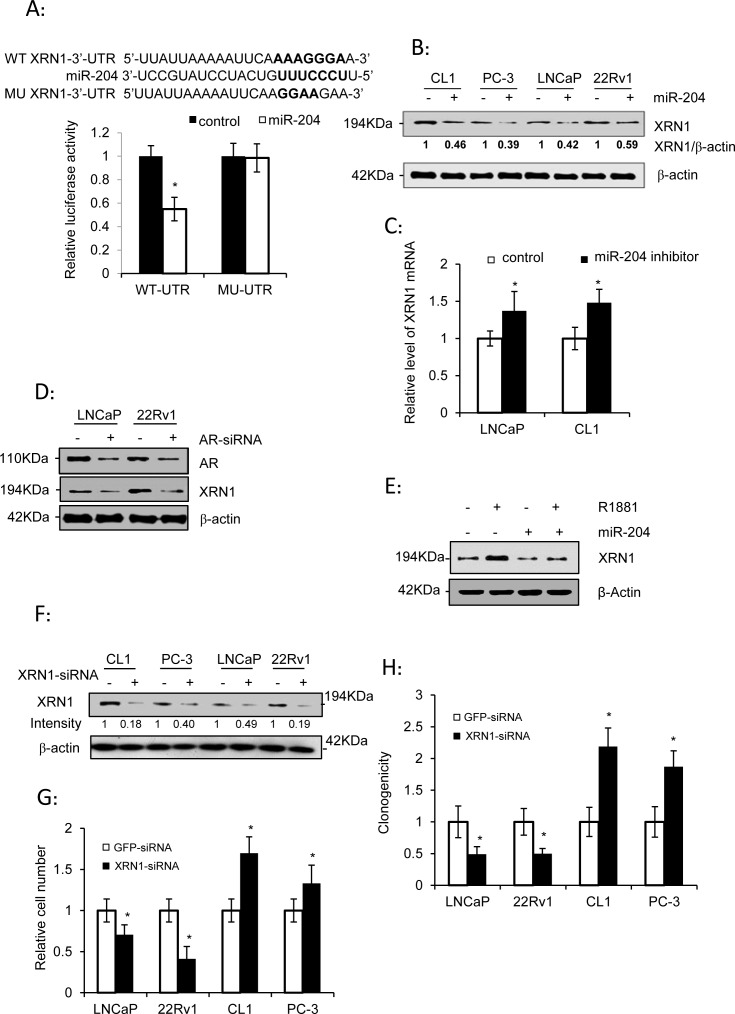
XRN1, as a miR-204 target, is a dual regulator of PCa cell growth (A) Luciferase assay of the reporter gene with wild-type (WT) or mutant (MU) 3′-UTR of XRN1 in LNCaP cells infected with or without miR-204-expressing lentivirus. (B) Western blot analysis of XRN1 expression in PCa cells in the presence of ectopic expression of miR-204 as indicated. (C) Levels of XRN1 mRNA in LNCaP and CL1 cells transfected with the miR-204 inhibitor or control oligonucleotides. (D) Western blot analysis of XRN1 expression in PAC cells with knockdown of AR. LNCaP and 22Rv1 cells were transfected with AR-siRNA or control RNA duplex. (E) Western blot analysis of the effect of miR-204 overexpression on regulation of XRN1 expression by androgen in LNCaP cells. (F) Western blot analysis of XRN1 in PCa cells transfected with XRN1 siRNA. (G and H) Effect of silencing of XRN1 on cell growth (G) and clonogenicity (H) of PCa cells. The data were obtained from at least three independent experiments, and the values are shown as the mean ± SEM; * p< 0.05; **p<0.01.

XRN1 (5′-3′ exoribonuclease 1) was inferred as a potential target of miR-204 *via* the algorithms of TargetScan 5.2 (http://targetscan.org/), PicTar (http://pictar.mdc-berlin.de/), and DIANA-microT v3.0 (http://diana.cslab.ece.ntua.gr/microT/). To validate it, a luciferase reporter construct was generated by cloning a 562-bp-long 3′-UTR of XRN1 mRNA downstream of the Renilla luciferase gene. Subsequently, our assay indicated that the luciferase activity in this reporter was inhibited by 47.8% in LNCaP cells (Fig. 3A). Futhermore, the mutations introduced to the miR-204-pairing sequence in 3-UTR of XRN1 almost reversed the inhibition of luciferase activity by miR-204 (Fig. 3A), indicating that miR-204 directly targeted the 3′-UTR of XRN1. In support of this, ectopic expression of miR-204 lowered the level of XRN1 protein in all the PCa cell lines tested (Fig. 3B), whereas introduction of the miR-204 inhibitor raised level of XRN1mRNA (Fig. 3C), suggesting that miR-204 is a repressor of XRN1 expression in PCa cells.

Given that miR-204 suppressed XRN1 expression (Fig. 3B), it suggests that AR might up-regulate XRN1 expression *via* its inhibitory effect on miR-204 expression (Fig. 1B). Consistent with this, introduction of AR-siRNA decreased XRN1 expression in LNCaP and 22Rv1 cells (Fig. 3D). Moreover, we detected that R1881 raised level of XRN1 in LNCaP cells, but the up-regulation was significantly blocked in the cells that overexpressed miR-204 (Fig. 3E). Taken together, these results indicated the presence of AR-miR-204-XRN1 signaling axis in PCa cells.

Finally, knocking down XRN1 (Fig. 3F) inhibited the growth of LNCaP and 22Rv1 cells (Fig. 3G), but increased the growth of CL1 and PC-3 cells (Fig. 3G). Similarly, while XRN1 knockdown significantly inhibited the colony-forming capacity of LNCaP and 22Rv1 cells, it instead increased the colony-formation of CL1 and PC-3 cells (Fig. 3H). These results indicated that silencing of XRN1 recapitulates the dual yet opposite function of miR-204 in mediating growth of different PCa cells.

### Androgen down-regulates miR-204 but up-regulates XRN1 in rat ventral prostate

**Figure 4:**
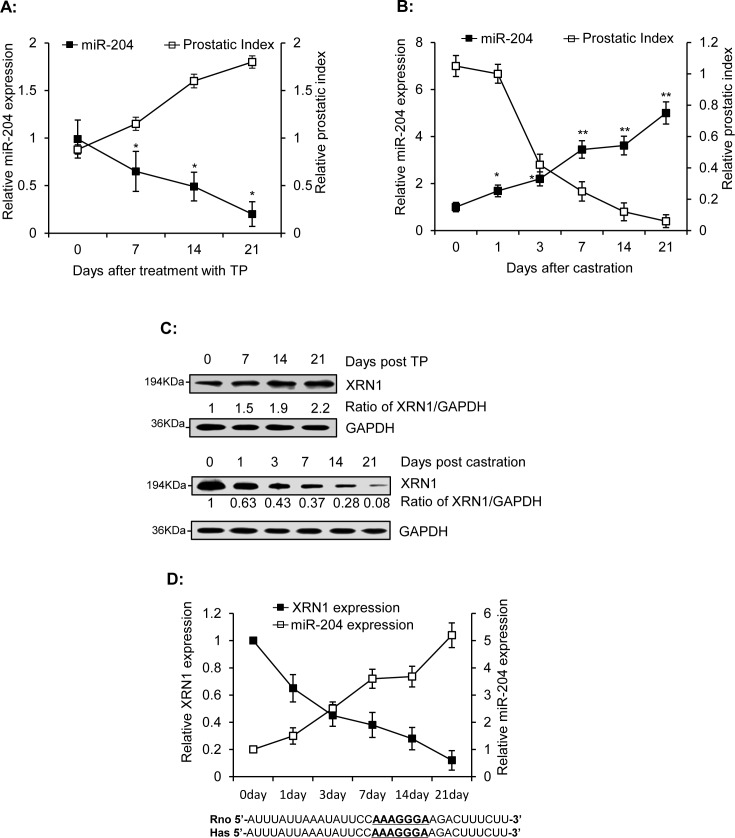
Inverse correlation of miR-204 and XRN1 expression in the ventral prostates of rats Male rats were injected with TP (s.c., 25mg/kg/day) for 1–3 weeks. Ventral prostates were removed from three rats at the indicated time and were used to measure levels of miR-204 using RT-qPCR (A) and XRN1 using immunoblotting analysis (C). Ventral prostates were isolated from the three castrated rats at various times after castration, as indicated, and were used to monitor miR-204 expression (B) and for immunoblotting analysis of XRN1 expression (C). The data were obtained from three independent assays (Bar, mean± SEM; * p<0.05; ** p<0.01, n=3). (D) Inverse correlation between miR-204 and XRN1 protein in ventral prostates of castrated rats*. Bottom,* The 33-bp sequences in the 3′-UTRs of rat XRN1 mRNA (from nt3731 to nt3763) and human XRN1 mRNA (from nt4619 to nt4651) including the base-pairs (underlined) complementary to seed sequence of miR-204.

We further measured levels of miR-204 in ventral prostates of rats that were injected with testosterone propionate (TP) (Fig. 4A) or castrated (Fig. 4B). Our results showed that miR-204 gradually decreased as the prostate index (gross weight of prostate/weight of whole animal ×100%) increased (Fig. 4A). However, castration dramatically increased expression of miR-204 (Fig. 4B). These results indicated that miR-204 is down-regulated by androgen in ventral prostates of rats. In contrast to miR-204, XRN1 was induced by androgen but inhibited by castration in rat ventral prostates (Fig. 4C). Given that XRN1 is a direct target of miR-204 (Fig. 3) and a miR-204-paring sequence was also identified in the 3′-UTR of rat XRN1 mRNA (Fig. 4D), the inverse relationship between miR-204 and XRN1 expression in ventral prostate of castrated rats (Fig. 4D) provided an evidence strongly supporting the presence of AR-miR-204-XRN1 axis *in vivo*.

### miR-204 and XRN1-siRNA repress AR expression and exhibit an dual regulation on key regulators of cell cycle progression in different PCa cells

**Figure 5:**
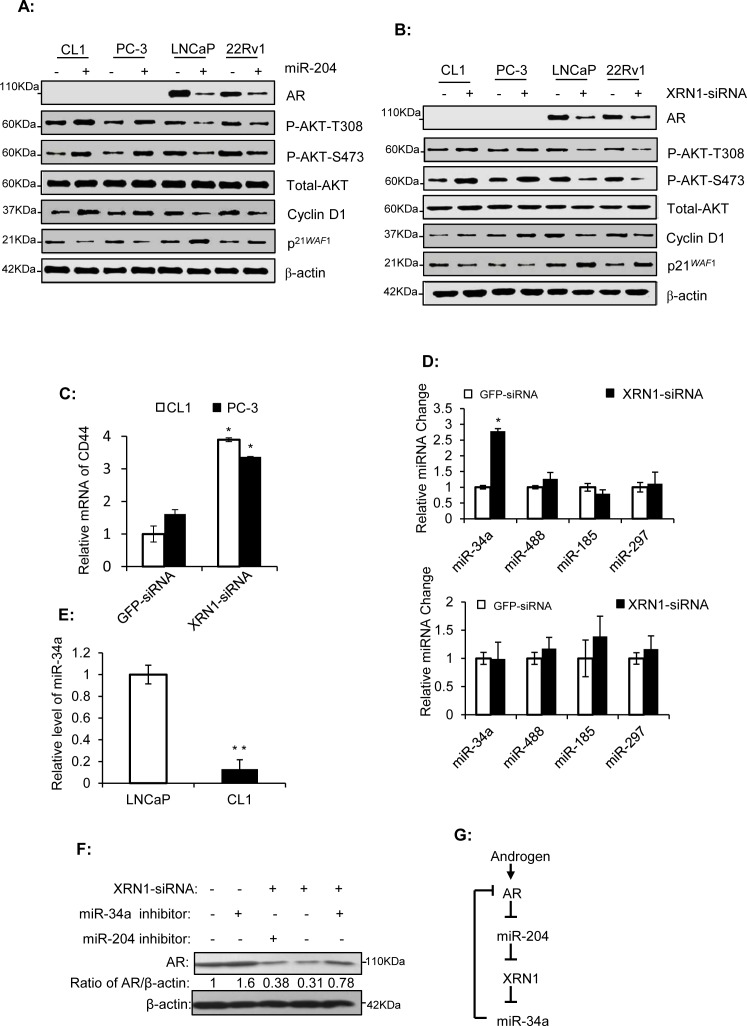
miR-204 and XRN1 regulate AR expression, and miR-34a is a XRN1 target that down-regulates AR (A-B) Western blot analyses of PCa cells infected with miR-204-expressing virus or transfected with XRN1-siRNA. (C and E) RT-PCR assays of CD44 and miR-34a in different PCa cells as indicated. (D) RT-qPCR assays of four AR-targeting miRNAs in LNCaP (*top*) and CL-1 (*bottom*) cells with XRN1 knockdown. Shown are mean values ± SEM. *P < 0.05; **P < 0.01. n=3. (F) Western blot analysis of the effect of miR-34a inhibitor on XRN1-siRNA-induced down-regulation of AR in LNCaP cells. (G) Schematic representation of the proposed AR/miR-204/XRN1/miR-34a feedback loop. The activation of the loop by androgen induces an up-regulation of AR signaling. The modulation is advantageous for development of aggressive phenotype of PAC.

Western blotting analysis showed that miR-204 and XRN-siRNA inhibited AR expression in the two PAC cell lines (Fig. 5A and B). Androgen was reported to inhibit the transcription of p21*^WAF1^*, an inhibitor of cell cycle progression in LNCaP cells [35]. Consistent with this, miR-204 and XRN-siRNA also increased levels of p21*^WAF1^* in two PAC cell lines (Fig. 5A and B). These results strongly suggested that down-regulation of AR by miR-204 or XRN-siRNA generates profound downstream signaling changes. In contrast to the response of p21*^WAF1^* to miR-204 and XRN-siRNA, Cyclin D1 and Akt phosphorylation (both at T308 and S473) showed a significant up-regulation in the NEPC cell lines, but a down-regulation in the PAC cell lines overexpressing miR-204 (Fig. 5A and B). These results revealed that the dual function of miR-204/XRN1 axis is closely associated with its dual modulation of expression of these key regulators of cell cycle progression. Finally, we studied effect of XRN1 knockdown on expression of CD44, the feature of NEPC cells [8-10]. Our results showed that XRN1-siRNA increased CD44 expression in both CL1 and PC-3 cells (Fig. 5C), suggesting that by down-regulating CD44 expression XRN1 could be a potential suppressor of NE phenotype of PCa.

### XRN1 induces AR expression via its downstream effector miR-34a

XRN1 is an exoribonuclease that participates in the degradation of mRNAs and miRs [36]. A number of miRs target AR [37], raising the possibility that XRN1 may, through degrading these miRs, maintain AR expression. To test this, we examined the impact of XRN1-siRNA on levels of four reported AR-targeting miRs (i.e. miR-34a,-488,-185,-297) in LNCaP and CL-1 cell lines. Our results showed that one of them, i.e, miR-34a was significantly increased by XRN1-siRNA (~2.78 folds) in LNCaP cells but not in CL-1 cells (Fig. 5D). Our result also showed that level of miR-34a in CL-1 cells is approximately 14.7% of that in LNCaP cells (Fig. 5E). This low level of miR-34a in CL-1 cells might be below the threshold needed for the exoribonuclease activity of XRN1, which may account for ineffectiveness of XRN1 on miR-34a expression in this cell line.

Given AR expression is inhibited by miR-34a [37, 38] and miR-34a expression is inhibited by XRN1 (Fig. 5D), it is expected that knockdown of XRN1 might reduce AR expression *via* miR-34a expression. Consistent with this, AR expression was induced moderately in LNCaP cells transfected with the miR-34a inhibitor when compared to its control (Fig. 5F), supporting that miR-34a is an AR-targeting miRNA [37]. In addition, AR expression in LNCaP cells transfected with XRN1-siRNA alone was approximately 31% of that in the cells transfected with GFP-siRNA, but approximately 78% of that in cells co-transfected with XRN1-siRNA and the miR-34a inhibitor (Fig. 5F). In contrast to the miR-34a inhibitor, the miR-204 inhibitor marginally changed the AR expression after it was co-transfected into LNCaP cells with XRN1-siRNA. Taken together, our results indicated that XRN1, *via* miR-34a, positively regulated AR expression. Given that miR-204 and XRN1 are regulated by AR (Figs. 1 and 3), these results established an AR-miR-204-XRN1-miR-34a feedback loop functionally active in PAC cells (Fig. 5G).

### Expression of miR-204 and XRN1 is down- and up-regulated in human PCa specimens, respectively

**Figure 6:**
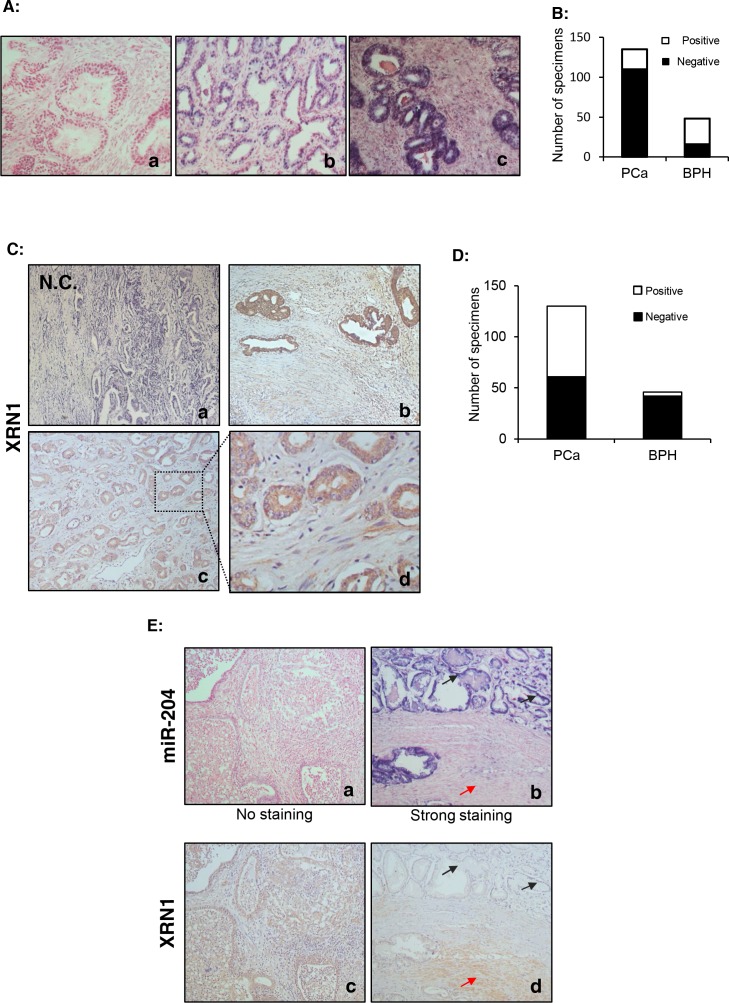
miR-204 is down-regulated but XRN is up-regulated in primary PCa specimens (A) A representative LNA-ISH of miR-204 in clinical PCa specimens. A moderate/strong miR-204 staining in the epithelium of a PCa specimen (b) and a BPH specimen (c). Notably, miR-204 was hardly observed in the stroma of the samples. (a) the negative control. (B) Summary of the numbers of PCa and BPH specimens with positive or negative expression of miR-204. (C) Representative IHC staining of XRN1 in PCa specimens in TMA, The framed area in (c) is shown with increased magnification in (d). (a) the negative control. (D) Summary of the numbers of PCa and BPH specimens with positive or negative XRN1 staining. (D) Inverse association of expression of miR-204 and XRN1 in PCa. Shown are IHC of two adjacent pairs (a/c & b/d) of TMA sections. *black arrow*, epithelium. *red arrow*, stroma.

To further understand the pathological relevance of miR-204 and XRN1 in PCa, we performed the LNA-ISH analysis and IHC to measure expression of miR-204 and XRN1in PCa specimens mounted on TMA slices. Our results showed that miR-204 is primarily expressed in the epithelium of PCa and BPH (Fig. 6A). Furthermore, we found that miR-204 expression was significantly down-regulated in PCa compared to BPH specimens. The positive rate of miR-204 expression was 66.7% (32/48) in BPH specimens *vs* 18.5% (25/135) in PCa specimens (p<0.0001) (Fig. 6B).

IHC staining indicated that XRN1 expression was primarily localized in the cytoplasm of luminal glandular cells and stromal cells in both BPH and PCa (Fig. 6C). XRN1 expression was positive in the epithelium in 53.1% (69/130) of PCa specimens. However, epithelial staining only detected XRN1 expression in 8.7% (4/46) of the BPH specimens (Fig. 6D). Additionally, stromal XRN1 expression was detected in 26.9% (29/108) of the PCa specimens and 8.7% (4/46) of the BPH specimens. The cellular localization of XRN1 in the PCa specimens was not significantly different from in the BPH specimens. Together, these results demonstrated that PCa has a higher level of XRN1 expression than BPH.

We further used two adjacent consecutive TMA sections to investigate whether there is an inverse relationship between the expression of miR-204 and XRN1 in PCa specimens. The results in Fig. 6E (a and c) indicated a negative miR-204 expression with a strong XRN1 expression in the same PCa specimen. In another PCa specimen, whereas we observed a strong staining of miR-204 in luminal epithelial cells, a strong expression of XRN1 was detected in the stroma with almost absent expression in luminal epithelial cells (Fig. 6E-d). These observations indicated that there is an inverse correlation between miR-204 and XRN1 expression in these specimens, supporting that miR-204 is a negative regulator of XRN1 in a subgroup of PCa patients.

### Positive epithelial XRN1 expression is associated with high levels of serum PSA in PCa patients

As shown in Table 1, expression of XRN1 and miR-204 did not show a significant association of with the pathological stage, Gleason score and recurrence. However, 63.8% (37/58) of patients with high PSA level (>17.8 ng/L, the median level in the patients analyzed) were found to be XRN1-positvie. The positive rate of XRN1 expression was only 44.8% (26/58) in patients with low PSA level (≤17.8 ng/L) (Table 1). This difference is statistically significant (p=0.04), suggesting that there is a correlation between epithelial XRN1 expression and serum PSA levels in PCa patients.

**Table 1:** Expression XRN1 and miR-204 across clinicopathological parameters

	XRN1 expression			miR-204 expression		
	Positive N (%)	Negative N (%)	*p* Value	Positive N (%)	Negative N (%)	*p* Value
Pathological stage			0.918			0.51
pT2–pT3a	57 (53.3)	48 (46.7)		14 (15.2)	78 (84.8)	
pT3b	9 (52.9)	8 (47.1)		3 (17.6)	14 (82.4)	
Recurrence			0.936			0.981
Positive	39 (54.2)	33 (45.8)		10 (58.8)	7 (41.2)	
Negative	28 (54.9)	23 (45.1)		55 (59.1)	38 (40.9)	
Gleason scores			0.455			0.286
7 or Greater	17 (60.7)	11 (39.3)		13 (18.3)	58 (81.7)	
6 or Less	49 (52.7)	44 (47.3)		4 (10.5)	34 (89.5)	
Serum PSA			0.04			0.791
≤Median	26 (44.8)	32 (55.2)		9 (17.3)	43 (82.7)	
>Median	37 (63.8)	21 (36.2)		8 (15.4)	44 (84.6)	

### Expression of miR-204 affects association of XRN1 expression with clinicopathological characteristics of PCa

Given XRN1 was validated as a miR-204 target, we further analyzed whether epithelial XRN1 expression was inversely associated with miR-204 expression in patient samples. As shown in Supplementary Table 4, we did not observe a significant inverse correlation in the total 171 clinical samples (BPH plus PCa). However, the correlation coefficient was −0.304 (p<0.05) in the 45 BPH specimens, indicating the presence of inverse association between expression of epithelial XRN1 and miR-204 in BPH. We did not observe an inverse correlation in the group of PCa patients. However, a weak but significant (coefficient=−0.269; p=0.021) inverse correlation was observed in PCa specimens only with high Gleason scores (N=74) (Supplementary Table 4). The inverse correlation was higher (coefficient=−0.533; p=0.028) in this subgroup without recurrence (N=20) (Supplementary Table 4). Taken together, these results suggest that miR-204 is the important regulator of XRN1 expression in the subgroup of poorly differentiated PCa which did not recur after ADT.

Finally, we also analyzed the association between XRN1 and clinicopathological parameters in the specimens with and without miR-204 expression (Table 2). Positive XRN1 expression was associated with higher Gleason scores only in miR-204 negative patients (N=99). By contrast, the association was not observed when the total PCa specimens (N=121) were used for the analysis (Table 1). In addition, positive XRN1 expression was inversely associated with recurrence among miR-204 positive patients (N=22), but not in miR-204-negative patients (Table 2), implying that XRN1 inhibits recurrence in miR-204 positive patients. The results strongly suggested that XRN1 has a different prognostic value in clinical PCa depending on miR-204 expression.

**Table 2:** miR-204 modification of the correlation of XRN1 expression with clinicopathological parameters

			XRN1 positive	XRN1 negative	p Value	OR	95%CI	
							lower	upper
miR-204 (−)	Pathological stage							
	pT2–pT3a		42(48.8%)	44(51.2%)	0.936	0.955	0.308	2.954
	pT3b		7(50.0%)	7(50.0%)				
	Recurrence							
	positive		32(53.3%)	28(46.7%)	0.352	1.46	0.657	3.245
	negative		18(43.9%)	23(56.1%)				
	Gleason score							
	7 or Greater		35(58.3%)	25(41.7%)	0.029	2.5	1.088	5.742
	6 or Less		14(35.9%)	25(64.1%)				
	PSA							
	≤17.8		18(39.1%)	28(60.9%)	0.063	0.459	0.201	1.047
	>17.8		28(58.3%)	20(41.7%)				
miR-204 (+)	Pathological stage							
	pT2–pT3a		15(78.9%)	4(21.1%)	1	1.875	0.134	26.32
	pT3b		2(66.7%)	1(33.3%)				
	Recurrence							
	positive		7(58.3%)	5(41.7%)	0.04	0.14	0.013	1.474
	negative		10(100.0%)	0(0.0%)				
	Gleason scores							
	7 or Greater		9(64.3%)	5(35.7%)	0.115	0.225	0.21	2.356
	6 or Less		8(100.0%)	0(0.0%)				
	PSA							
	≤17.8		8(66.7%)	4(33.3%)	0.323	0.222	0.2	2.424
	>17.8		9(90.0%)	1(10.0%)				

## DISCUSSION

In the present study, we demonstrated the presence of an AR-miR-204-XRN1 axis both in the cultured PCa cells (Figs. 1 and 3) and in ventral prostates of rat (Fig. 4). We showed that miR-204 has a tumor suppressive function in PAC cells, but acts as an oncomiR in NEPC cells (Fig. 2). Such a dual yet opposite regulatory function of miR-204 was also observed by our study of miR-204 target XRN1 (Fig. 3). Furthermore, the dual regulatory function of miR-204/XRN1 axis was demonstrated by its dual-regulation of some key regulators of cell cycle, including pAKT, p21*^WAF1^* and Cyclin D1 (Fig. 5A and B). To the best of our knowledge, this is the first report showing that a single miRNA has the dual yet opposite regulatory function in the two types of PCa cell models. In addition, We also demonstrated that XRN1 selectively down-regulates expression of miR-34a, an AR-targeting micro-RNA (Fig. 5D), and that inactivation of miR-34a reduces expression of AR (Fig. 5F) [39] and increases aggressiveness of PAC cells [39]. Therefore, our analysis further expands AR-miR-204-XRN1 axis to AR-miR-204-XRN1-miR-34a feedback loop. In this loop, androgen up-regulates XRN1, by repressing miR-204 expression, while XRN1 raises AR expression by reducing expression of miR-34a (Fig. 5G). This loop forms a positively regulatory feedback for function of AR signaling in PAC cells and NEPC cells, and may represent a novel mechanism contributing to the dual yet opposite role of AR signaling in PCa progression [40].

Thus far, the molecular mechanism involved in development of NEPC remains unclear. For those SCNCs that arise from NED of PAC, it is believed that the rapidly dividing NEPC have completely overtaken the slowly growing adenocarcinoma, resulting in histological appearance of a pure SCNC. Therefore, it is possible that during the development of ADT-driven SCNC, ADT induces an up-regulated expression of miR-204, which, in turn, reduces AR expression, and eventually enables certain prostate tumor clones to assume a more NE phenotype. In addition, given our results strongly suggest that the dual function of miR-204 is decided by the status of AR expression in PCa cells, the adaptive shift of miR-204 towards oncomiR could be another important step following weakening or losing of AR signaling during the development of SCNC. Notably, the biological aggressiveness of prostatic NE tumor cells is probably further promoted by a sharp down-regulation of XRN1 expression resulted from androgen-deprivation (Fig. 4C). Moreover, since CD44 knockdown represses Akt phosphorylation and growth of PC-3 cells [41], inhibition of CD44 expression by XRN1 (Fig. 5C) is likely critical for XRN1 tumor suppressive function in NEPC cells. Finally, it should be noted that down-regulation of miR-34a by XRN1 might contribute to PCa progression independent of AR, since miR-34a can act as a tumor suppressor through directly inhibiting CD44 in tumorigenic and metastatic PCa stem cells [42].

Function of AR/miR-204/XRN1/miR-34a loop could be regulated by many factors associated with PCa progression. For example, miR-34a expression is activated transcriptionally by the tumor suppressor p53 [43]. Given that *TP53* is mutated in majority of NE tumor cells, which is important for progression of SCNC [44], it may help us to understand why compared with LNCaP cells that express wild-type p53, p53-null CL1 and PC-3 cells [33, 45] express low levels of miR-34a (Fig. 5E) [46]. Because of this, there might be a mutually functional regulation between XRN1 and p53 through miR-34a in PCa cells. Similarly, since CD44 is a target of miR-34a [42], high levels of CD44 expression in NEPC cells [8-10] are probably resulted from low levels of miR-34a. Furthermore, given low expression of miR-34a is not sensitive to knockdown of XRN1 in CL-1 cells (Fig. 5D), inhibition of CD44 expression by XRN1 (Fig. 5C) suggests that XRN1 regulates CD44 expression in the way independent of miR-34a in NEPC cells.

miR-204 has been shown to down-regulated in a number of cancers [47-49], consistent with its potential tumor suppressive role. However, miR-204 expression was also shown high in some cancers such as melanomas [50]. An elevated expression of miR-204 was also previously reported in PCa, in which only 5 PCa specimens without pathological information were used [51]. Here we used a TMA that included 135 PCa specimens to analyze the association between miR-204 expression and PCa, and our result showed that lower levels of miR-204 (Fig. 6B) and higher levels of XRN1 (Fig. 6D) in primary PCa than that in the control samples, respectively. Moreover, an association of positive XRN1 expression in the epithelium with high serum PSA levels in PCa patients (Table 1) was observed, which is consistent with our results that XRN1 plays a role in maintaining expression of AR (Fig. 5B), presumably controlling AR target PSA expression. Therefore, our results indicate an important prognostic role of XRN1 in PCa. Finally, our preliminary analysis also suggested the presence of recurrence-inhibitory role of epithelial XRN1 (Table 2). Because of the limited numbers of patients, this result needs to be further validated by using a larger number of specimens. Nevertheless, our results so far support that XRN1 may repress recurrence *via* its tumor suppressive activity in NEPC cells. In this respect, it will be important to further examine whether an inverse association between expression of XRN1 and NE markers exists in CRPC and SCNC in the future.

In conclusion, we have demonstrated that AR-miR-204-XRN1-miR-34a feedback loop (Fig. 5F) plays an important role in regulating growth of PAC cells. In contrast to its tumor suppressive role in PAC cells, miR-204, by targeting XRN1, functions as an oncomiR in NEPC cells. These findings have not only provided a novel mechanistic insight into ADT-induced NED, but also established a strong rationale for us to develop a cell type-specific strategy targeting miR-204 as a novel therapeutic approach against prostatic SCNC.

## MATERIALS AND METHODS

### Cell culture infection and transfection

The human PCa cell lines LNCaP, 22Rv1 and PC-3 cells were obtained from ATCC (Manassas, VA). LNCaP and CL1 cells were grown as described by Tso et al [23]. PC-3 and 22Rv1 cells were cultured in RPMI 1640 medium supplemented with 10% fetal bovine serum (FBS) at 37° C in a humidified air atmosphere with 5% CO_2_. miR-34a, miR-204 (RiboBio, Guangzhou), AR-siRNA and XRN1-siRNA (GenePharma, Shanghai) were transfected into cells using Lipofectamine 2000 (Invitrogen). Transfection of AR expression construct (pCMV-hAR) or its empty control vector into PC-3 cells and treatment of these cells with R1881 (Sigma, USA) were performed as previously described [24]. miR-204-expressing recombinant lentivirus and its control virus were purchased from Kangchen Bio-tech (Shanghai, China). The delivery of miR-204 by viral infection was performed according to the manufacturer's instructions.

### Cell number determination, colony formation assay, luciferase reporter assays, immunoblotting, xenograft analysis and qRT-PCR

These analyses were carried out as described previously [25, 26]. Bulge-Loop TMmiRNA qPCR primer sets (RiBoBio, Guangzhou, China) were used to measure levels of miR-34a, miR-185, miR-297 and miR-488. The sequences of primers used for PCR in this study are listed in Supplementary Table 1.

### Immunohistochemistry (IHC) and locked nucleic acid-in situ hybridization (LNA-ISH) in PCa tissue microarray (TMA)

The TMA-based experiments were performed according to published elsewhere [26, 27]. The sequence of LNA probes for miR-204 (Exiqon, Vedbaek, Denmark) was: 5′DIG-AGGCATAGGATGACAAAGGGAA-3′DIG. ISH-scores and IHC-scores were generated as previously described [28].

### Experimental benign prostatic hypoplasia (BPH)

The rats were injected with testosterone propionate (TP) (Sigma, USA) for generation of experimental BPH or castrated, as previously described [29].

### Statistics

Statistical significance was analyzed by student's t-test and expressed as a *P* value. For analysis of the association between XRN1 expression or miR-204 expression and clinicopathological parameters, chi-square test was performed. For evaluation of the correlation between XRN1 expression and miR-204 expression in clinical prostate specimens, linear regression analysis was carried out.

## SUPPLEMENTARY MATERIAL TABLES



## References

[1] Siegel R, Naishadham D, Jemal A (2012). Cancer statistics, 2012. CA Cancer J Clin.

[2] Chen CD, Welsbie DS, Tran C, Baek SH, Chen R, Vessella R, Rosenfeld MG, Sawyers CL (2004). Molecular determinants of resistance to antiandrogen therapy. Nat Med.

[3] Isaacs JT, Isaacs WB (2004). Androgen receptor outwits prostate cancer drugs. Nat Med.

[4] Wang Q, Li W, Liu XS, Carroll JS, Janne OA, Keeton EK, Chinnaiyan AM, Pienta KJ, Brown M (2007). A hierarchical network of transcription factors governs androgen receptor-dependent prostate cancer growth. Mol Cell.

[5] Shen MM, Abate-Shen C (2010). Molecular genetics of prostate cancer: new prospects for old challenges. Genes & development.

[6] Vashchenko N, Abrahamsson PA (2005). Neuroendocrine differentiation in prostate cancer: implications for new treatment modalities. European urology.

[7] Grignon DJ (2004). Unusual subtypes of prostate cancer. Modern pathology: an official journal of the United States and Canadian Academy of Pathology, Inc.

[8] Tai S, Sun Y, Squires JM, Zhang H, Oh WK, Liang CZ, Huang J (2011). PC3 is a cell line characteristic of prostatic small cell carcinoma. Prostate.

[9] Palapattu GS, Wu C, Silvers CR, Martin HB, Williams K, Salamone L, Bushnell T, Huang LS, Yang Q, Huang J (2009). Selective expression of CD44, a putative prostate cancer stem cell marker, in neuroendocrine tumor cells of human prostate cancer. Prostate.

[10] Simon RA, di Sant'Agnese PA, Huang LS, Xu H, Yao JL, Yang Q, Liang S, Liu J, Yu R, Cheng L, Oh WK, Palapattu GS, Wei J, Huang J (2009). CD44 expression is a feature of prostatic small cell carcinoma and distinguishes it from its mimickers. Hum Pathol.

[11] Miyoshi Y, Uemura H, Kitami K, Satomi Y, Kubota Y, Hosaka M (2001). Neuroendocrine differentiated small cell carcinoma presenting as recurrent prostate cancer after androgen deprivation therapy. BJU Int.

[12] Cindolo L, Cantile M, Vacherot F, Terry S, de la Taille A (2007). Neuroendocrine differentiation in prostate cancer: from lab to bedside. Urol Int.

[13] Jiborn T, Bjartell A, Abrahamsson PA (1998). Neuroendocrine differentiation in prostatic carcinoma during hormonal treatment. Urology.

[14] Hirano D, Okada Y, Minei S, Takimoto Y, Nemoto N (2004). Neuroendocrine differentiation in hormone refractory prostate cancer following androgen deprivation therapy. Eur Urol.

[15] Wright ME, Tsai MJ, Aebersold R (2003). Androgen receptor represses the neuroendocrine transdifferentiation process in prostate cancer cells. Mol Endocrinol.

[16] Yuan TC, Veeramani S, Lin FF, Kondrikou D, Zelivianski S, Igawa T, Karan D, Batra SK, Lin MF (2006). Androgen deprivation induces human prostate epithelial neuroendocrine differentiation of androgen-sensitive LNCaP cells. Endocr Relat Cancer.

[17] Jin RJ, Wang Y, Masumori N, Ishii K, Tsukamoto T, Shappell SB, Hayward SW, Kasper S, Matusik RJ (2004). NE-10 neuroendocrine cancer promotes the LNCaP xenograft growth in castrated mice. Cancer research.

[18] Bonkhoff H, Stein U, Remberger K (1995). Endocrine-paracrine cell types in the prostate and prostatic adenocarcinoma are postmitotic cells. Hum Pathol.

[19] Fletcher CE, Dart DA, Sita-Lumsden A, Cheng H, Rennie PS, Bevan CL (2012). Androgen-regulated processing of the oncomir miR-27a, which targets Prohibitin in prostate cancer. Hum Mol Genet.

[20] Lin PC, Chiu YL, Banerjee S, Park K, Mosquera JM, Giannopoulou E, Alves P, Tewari AK, Gerstein MB, Beltran H, Melnick AM, Elemento O, Demichelis F, Rubin MA (2013). Epigenetic repression of miR-31 disrupts androgen receptor homeostasis and contributes to prostate cancer progression. Cancer Res.

[21] Mo W, Zhang J, Li X, Meng D, Gao Y, Yang S, Wan X, Zhou C, Guo F, Huang Y, Amente S, Avvedimento EV, Xie Y, Li Y (2013). Identification of novel AR-targeted microRNAs mediating androgen signalling through critical pathways to regulate cell viability in prostate cancer. PLoS One.

[22] Ribas J, Ni X, Haffner M, Wentzel EA, Salmasi AH, Chowdhury WH, Kudrolli TA, Yegnasubramanian S, Luo J, Rodriguez R, Mendell JT, Lupold SE (2009). miR-21: an androgen receptor-regulated microRNA that promotes hormone-dependent and hormone-independent prostate cancer growth. Cancer Res.

[23] Tso CL, McBride WH, Sun J, Patel B, Tsui KH, Paik SH, Gitlitz B, Caliliw R, van Ophoven A, Wu L, deKernion J, Belldegrun A (2000). Androgen deprivation induces selective outgrowth of aggressive hormone-refractory prostate cancer clones expressing distinct cellular and molecular properties not present in parental androgen-dependent cancer cells. Cancer J.

[24] Lin B, Wang J, Hong X, Yan X, Hwang D, Cho JH, Yi D, Utleg AG, Fang X, Schones DE, Zhao K, Omenn GS, Hood L (2009). Integrated expression profiling and ChIP-seq analyses of the growth inhibition response program of the androgen receptor. PLoS One.

[25] Talotta F, Cimmino A, Matarazzo MR, Casalino L, De Vita G, D'Esposito M, Di Lauro R, Verde P (2009). An autoregulatory loop mediated by miR-21 and PDCD4 controls the AP-1 activity in RAS transformation. Oncogene.

[26] Wang W, Li Y, Hong A, Wang J, Lin B, Li R (2009). NDRG3 is an androgen regulated and prostate enriched gene that promotes *in vitro* and in vivo prostate cancer cell growth. Int J Cancer.

[27] Li T, Li RS, Li YH, Zhong S, Chen YY, Zhang CM, Hu MM, Shen ZJ (2012). miR-21 as an independent biochemical recurrence predictor and potential therapeutic target for prostate cancer. J Urol.

[28] Soumaoro LT, Uetake H, Higuchi T, Takagi Y, Enomoto M, Sugihara K (2004). Cyclooxygenase-2 expression: a significant prognostic indicator for patients with colorectal cancer. Clin Cancer Res.

[29] di Salle E, Giudici D, Biagini L, Cominato C, Briatico G, Panzeri A (1995). Effects of 5 alpha-reductase inhibitors on intraprostatic androgens in the rat. J Steroid Biochem Mol Biol.

[30] Sramkoski RM, Pretlow TG, Giaconia JM, Pretlow TP, Schwartz S, Sy MS, Marengo SR, Rhim JS, Zhang D, Jacobberger JW (1999). A new human prostate carcinoma cell line, 22Rv1. *In Vitro* Cell Dev Biol Anim.

[31] Shen R, Dorai T, Szaboles M, Katz AE, Olsson CA, Buttyan R (1997). Transdifferentiation of cultured human prostate cancer cells to a neuroendocrine cell phenotype in a hormone-depleted medium. Urologic oncology.

[32] Pascal LE, Vencio RZ, Vessella RL, Ware CB, Vencio EF, Denyer G, Liu AY (2011). Lineage relationship of prostate cancer cell types based on gene expression. BMC Med Genomics.

[33] Lin B, White JT, Lu W, Xie T, Utleg AG, Yan X, Yi EC, Shannon P, Khrebtukova I, Lange PH, Goodlett DR, Zhou D, Vasicek TJ, Hood L (2005). Evidence for the presence of disease-perturbed networks in prostate cancer cells by genomic and proteomic analyses: a systems approach to disease. Cancer Res.

[34] Beltran H, Rickman DS, Park K, Chae SS, Sboner A, MacDonald TY, Wang Y, Sheikh KL, Terry S, Tagawa ST, Dhir R, Nelson JB, de la Taille A, Allory Y, Gerstein MB, Perner S (2012). Molecular characterization of neuroendocrine prostate cancer and identification of new drug targets. Cancer Discov.

[35] Lu S, Liu M, Epner DE, Tsai SY, Tsai MJ (1999). Androgen regulation of the cyclin-dependent kinase inhibitor p21 gene through an androgen response element in the proximal promoter. Mol Endocrinol.

[36] Bail S, Swerdel M, Liu H, Jiao X, Goff LA, Hart RP, Kiledjian M (2010). Differential regulation of microRNA stability. RNA.

[37] Ostling P, Leivonen SK, Aakula A, Kohonen P, Makela R, Hagman Z, Edsjo A, Kangaspeska S, Edgren H, Nicorici D, Bjartell A, Ceder Y, Perala M, Kallioniemi O (2011). Systematic analysis of microRNAs targeting the androgen receptor in prostate cancer cells. Cancer Res.

[38] Shi XB, Xue L, Ma AH, Tepper CG, Gandour-Edwards R, Kung HJ, Devere White RW (2012). Tumor suppressive miR-124 targets androgen receptor and inhibits proliferation of prostate cancer cells. Oncogene.

[39] Kashat M, Azzouz L, Sarkar SH, Kong D, Li Y, Sarkar FH (2012). Inactivation of AR and Notch-1 signaling by miR-34a attenuates prostate cancer aggressiveness. Am J Transl Res.

[40] Niu Y, Chang TM, Yeh S, Ma WL, Wang YZ, Chang C (2010). Differential androgen receptor signals in different cells explain why androgen-deprivation therapy of prostate cancer fails. Oncogene.

[41] Hao J, Madigan MC, Khatri A, Power CA, Hung TT, Beretov J, Chang L, Xiao W, Cozzi PJ, Graham PH, Kearsley JH, Li Y (2012). *In vitro* and *in vivo* prostate cancer metastasis and chemoresistance can be modulated by expression of either CD44 or CD147. PLoS One.

[42] Liu C, Kelnar K, Liu B, Chen X, Calhoun-Davis T, Li H, Patrawala L, Yan H, Jeter C, Honorio S, Wiggins JF, Bader AG, Fagin R, Brown D, Tang DG (2011). The microRNA miR-34a inhibits prostate cancer stem cells and metastasis by directly repressing CD44. Nat Med.

[43] He L, He X, Lim LP, de Stanchina E, Xuan Z, Liang Y, Xue W, Zender L, Magnus J, Ridzon D, Jackson AL, Linsley PS, Chen C, Lowe SW, Cleary MA, Hannon GJ (2007). A microRNA component of the p53 tumour suppressor network. Nature.

[44] Chen H, Sun Y, Wu C, Magyar CE, Li X, Cheng L, Yao JL, Shen S, Osunkoya AO, Liang C, Huang J (2012). Pathogenesis of prostatic small cell carcinoma involves the inactivation of the P53 pathway. Endocrine-related cancer.

[45] van Bokhoven A, Varella-Garcia M, Korch C, Johannes WU, Smith EE, Miller HL, Nordeen SK, Miller GJ, Lucia MS (2003). Molecular characterization of human prostate carcinoma cell lines. Prostate.

[46] Fujita Y, Kojima K, Hamada N, Ohhashi R, Akao Y, Nozawa Y, Deguchi T, Ito M (2008). Effects of miR-34a on cell growth and chemoresistance in prostate cancer PC3 cells. Biochem Biophys Res Commun.

[47] Chen L, Yan HX, Yang W, Hu L, Yu LX, Liu Q, Li L, Huang DD, Ding J, Shen F, Zhou WP, Wu MC, Wang HY (2009). The role of microRNA expression pattern in human intrahepatic cholangiocarcinoma. J Hepatol.

[48] Chung TK, Lau TS, Cheung TH, Yim SF, Lo KW, Siu NS, Chan LK, Yu MY, Kwong J, Doran G, Barroilhet LM, Ng AS, Wong RR, Wang VW, Mok SC, Smith DI (2012). Dysregulation of microRNA-204 mediates migration and invasion of endometrial cancer by regulating FOXC1. Int J Cancer.

[49] Imam JS, Plyler JR, Bansal H, Prajapati S, Bansal S, Rebeles J, Chen HI, Chang YF, Panneerdoss S, Zoghi B, Buddavarapu KC, Broaddus R, Hornsby P, Tomlinson G, Dome J, Vadlamudi RK (2012). Genomic loss of tumor suppressor miRNA-204 promotes cancer cell migration and invasion by activating AKT/mTOR/Rac1 signaling and actin reorganization. PloS one.

[50] Jukic DM, Rao UN, Kelly L, Skaf JS, Drogowski LM, Kirkwood JM, Panelli MC (2010). Microrna profiling analysis of differences between the melanoma of young adults and older adults. J Transl Med.

[51] Turner DP, Findlay VJ, Moussa O, Semenchenko VI, Watson PM, LaRue AC, Desouki MM, Fraig M, Watson DK (2011). Mechanisms and functional consequences of PDEF protein expression loss during prostate cancer progression. Prostate.

