# Supplementary material for: A dual yet opposite growth-regulating function of miR-204 and its target XRN1 in prostate adenocarcinoma cells and neuroendocrine-like prostate cancer cells
Source: Oncotarget. 2015 Mar 8;6(10):7686–700. doi: 10.18632/oncotarget.3480 (PMC4480709; doi:10.18632/oncotarget.3480)
Supplement: Supplementary file 1 [file oncotarget-06-7686-s001.pdf]

A dual yet opposite growth-regulating function of miR-204 and its target XRN1 in prostate adenocarcinoma cells and neuroendocrine-like prostate cancer cells

Supplementary Material

Supplementary Table 1

Raw data (mean spot intensities) of miRNA arrays hybridized with cDNA probes from LNCaP cells treated with androgens (R1881, 1nM) and mock control (ethanol).

miRNA name	Mock Control (Ethanol)	Androgens (R1881, 1 nM)
hsa-let-7a	534	376
hsa-let-7a	444	304
hsa-let-7a	432	300
hsa-let-7a	494	336
hsa-let-7b	524	502
hsa-let-7b	591	563
hsa-let-7b	695	539
hsa-let-7b	567	459
hsa-let-7c	428	346
hsa-let-7c	421	332
hsa-let-7c	586	360
hsa-let-7c	471	342
hsa-let-7d	539	479
hsa-let-7d	536	468
hsa-let-7d	859	684
hsa-let-7d	880	702
hsa-let-7e	499	500
hsa-let-7e	519	504
hsa-let-7e	469	442

hsa-let-7e	400	355
hsa-let-7f	282	229
hsa-let-7f	311	266
hsa-let-7f	322	264
hsa-let-7f	329	277
hsa-let-7g	196	183
hsa-let-7g	200	174
hsa-let-7g	194	176
hsa-let-7g	198	182
hsa-let-7i	215	169
hsa-let-7i	215	175
hsa-let-7i	222	171
hsa-let-7i	218	177
hsa-miR-1	225	171
hsa-miR-1	225	165
hsa-miR-1	230	162
hsa-miR-1	215	168
hsa-miR-100	304	214
hsa-miR-100	311	222
hsa-miR-100	300	212
hsa-miR-100	317	226
hsa-miR-101	183	166
hsa-miR-101	181	163
hsa-miR-101	179	169
hsa-miR-101	180	162
hsa-miR-103	194	176

hsa-miR-103	205	181
hsa-miR-103	192	173
hsa-miR-103	196	185
hsa-miR-105	252	176
hsa-miR-105	247	176
hsa-miR-105	268	165
hsa-miR-105	256	167
hsa-miR-106a	219	199
hsa-miR-106a	209	184
hsa-miR-106a	253	187
hsa-miR-106a	210	191
hsa-miR-106b	272	217
hsa-miR-106b	299	233
hsa-miR-106b	307	233
hsa-miR-106b	339	228
hsa-miR-107	208	199
hsa-miR-107	209	195
hsa-miR-107	229	205
hsa-miR-107	235	214
hsa-miR-10a	209	181
hsa-miR-10a	206	170
hsa-miR-10a	210	167
hsa-miR-10a	212	176
hsa-miR-10b	180	164
hsa-miR-10b	184	164
hsa-miR-10b	185	164

hsa-miR-10b	183	161
hsa-miR-122a	231	167
hsa-miR-122a	248	184
hsa-miR-122a	254	207
hsa-miR-122a	238	166
hsa-miR-124a	193	175
hsa-miR-124a	190	176
hsa-miR-124a	189	178
hsa-miR-124a	190	179
hsa-miR-125a	294	366
hsa-miR-125a	307	373
hsa-miR-125a	311	346
hsa-miR-125a	310	338
hsa-miR-125b	669	405
hsa-miR-125b	545	343
hsa-miR-125b	564	339
hsa-miR-125b	669	375
hsa-miR-126	179	167
hsa-miR-126	178	173
hsa-miR-126	176	168
hsa-miR-126	181	169
hsa-miR-126*	179	162
hsa-miR-126*	179	164
hsa-miR-126*	247	190
hsa-miR-126*	186	167
hsa-miR-127	192	172

hsa-miR-127	205	171
hsa-miR-127	197	177
hsa-miR-127	197	175
hsa-miR-128a	240	172
hsa-miR-128a	239	168
hsa-miR-128a	244	168
hsa-miR-128a	243	169
hsa-miR-128b	243	166
hsa-miR-128b	242	165
hsa-miR-128b	236	161
hsa-miR-128b	242	168
hsa-miR-129	292	244
hsa-miR-129	294	239
hsa-miR-129	292	228
hsa-miR-129	323	252
hsa-miR-130a	198	229
hsa-miR-130a	224	252
hsa-miR-130a	219	232
hsa-miR-130a	200	213
hsa-miR-130b	230	206
hsa-miR-130b	237	224
hsa-miR-130b	247	221
hsa-miR-130b	234	211
hsa-miR-132	184	177
hsa-miR-132	200	170
hsa-miR-132	199	175

hsa-miR-132	196	170
hsa-miR-133a-133b	465	686
hsa-miR-133a-133b	494	639
hsa-miR-133a-133b	491	688
hsa-miR-133a-133b	507	649
hsa-miR-133b	605	836
hsa-miR-133b	689	818
hsa-miR-133b	681	777
hsa-miR-133b	661	737
hsa-miR-134	206	230
hsa-miR-134	207	228
hsa-miR-134	207	224
hsa-miR-134	217	211
hsa-miR-135a	232	160
hsa-miR-135a	231	168
hsa-miR-135a	242	164
hsa-miR-135a	243	164
hsa-miR-135b	190	162
hsa-miR-135b	234	173
hsa-miR-135b	205	163
hsa-miR-135b	197	167
hsa-miR-136	214	169
hsa-miR-136	212	168
hsa-miR-136	207	165
hsa-miR-136	213	167
hsa-miR-137	213	171

hsa-miR-137	220	172
hsa-miR-137	228	173
hsa-miR-137	226	168
hsa-miR-138	210	169
hsa-miR-138	235	171
hsa-miR-138	217	161
hsa-miR-138	223	167
hsa-miR-139	182	168
hsa-miR-139	188	164
hsa-miR-139	208	168
hsa-miR-139	187	166
hsa-miR-140	185	166
hsa-miR-140	184	162
hsa-miR-140	189	160
hsa-miR-140	187	166
hsa-miR-141	201	176
hsa-miR-141	230	182
hsa-miR-141	205	171
hsa-miR-141	208	170
hsa-miR-142-3p	187	164
hsa-miR-142-3p	192	157
hsa-miR-142-3p	185	169
hsa-miR-142-3p	200	164
hsa-miR-142-5p	178	165
hsa-miR-142-5p	181	172
hsa-miR-142-5p	198	166

hsa-miR-142-5p	185	170
hsa-miR-143	180	165
hsa-miR-143	189	176
hsa-miR-143	181	165
hsa-miR-143	179	173
hsa-miR-144	264	162
hsa-miR-144	266	172
hsa-miR-144	264	165
hsa-miR-144	277	174
hsa-miR-145	218	168
hsa-miR-145	226	167
hsa-miR-145	223	163
hsa-miR-145	211	168
hsa-miR-146a	192	171
hsa-miR-146a	212	177
hsa-miR-146a	191	169
hsa-miR-146a	217	176
hsa-miR-146a	189	163
hsa-miR-146a	222	172
hsa-miR-146a	189	163
hsa-miR-146a	222	171
hsa-miR-146b	189	161
hsa-miR-146b	190	167
hsa-miR-146b	189	162
hsa-miR-146b	199	166
hsa-miR-147	200	158

hsa-miR-147	205	164
hsa-miR-147	249	191
hsa-miR-147	210	168
hsa-miR-148a	211	177
hsa-miR-148a	201	179
hsa-miR-148a	218	188
hsa-miR-148a	216	177
hsa-miR-148b	210	175
hsa-miR-148b	213	174
hsa-miR-148b	213	176
hsa-miR-148b	217	172
hsa-miR-149	190	166
hsa-miR-149	189	170
hsa-miR-149	187	163
hsa-miR-149	189	170
hsa-miR-150	291	184
hsa-miR-150	295	182
hsa-miR-150	323	184
hsa-miR-150	284	181
hsa-miR-151	183	167
hsa-miR-151	188	174
hsa-miR-151	197	171
hsa-miR-151	199	171
hsa-miR-152	187	170
hsa-miR-152	188	168
hsa-miR-152	176	170

hsa-miR-152	189	168
hsa-miR-153	176	169
hsa-miR-153	178	178
hsa-miR-153	180	169
hsa-miR-153	184	170
hsa-miR-154	221	177
hsa-miR-154	210	165
hsa-miR-154	219	174
hsa-miR-154	223	166
hsa-miR-154*	225	178
hsa-miR-154*	216	173
hsa-miR-154*	219	180
hsa-miR-154*	219	171
hsa-miR-155	288	191
hsa-miR-155	283	179
hsa-miR-155	288	184
hsa-miR-155	291	191
hsa-miR-15a	269	178
hsa-miR-15a	276	177
hsa-miR-15a	279	178
hsa-miR-15a	275	176
hsa-miR-15b	256	170
hsa-miR-15b	253	175
hsa-miR-15b	271	171
hsa-miR-15b	275	171
hsa-miR-16	216	188

hsa-miR-16	222	193
hsa-miR-16	299	225
hsa-miR-16	271	212
hsa-miR-17-3p	185	174
hsa-miR-17-3p	224	221
hsa-miR-17-3p	210	171
hsa-miR-17-3p	201	173
hsa-miR-17-5p	195	189
hsa-miR-17-5p	197	195
hsa-miR-17-5p	238	215
hsa-miR-17-5p	241	219
hsa-miR-181a	215	166
hsa-miR-181a	212	171
hsa-miR-181a	224	171
hsa-miR-181a	233	171
hsa-miR-181a*	180	174
hsa-miR-181a*	182	171
hsa-miR-181a*	181	171
hsa-miR-181a*	182	177
hsa-miR-181b	227	171
hsa-miR-181b	225	170
hsa-miR-181b	228	173
hsa-miR-181b	232	176
hsa-miR-181c	185	163
hsa-miR-181c	190	163
hsa-miR-181c	189	167

hsa-miR-181c	190	171
hsa-miR-181d	194	170
hsa-miR-181d	203	182
hsa-miR-181d	198	171
hsa-miR-181d	190	177
hsa-miR-182	207	174
hsa-miR-182	210	171
hsa-miR-182	215	169
hsa-miR-182	209	170
hsa-miR-182*	213	182
hsa-miR-182*	236	188
hsa-miR-182*	224	172
hsa-miR-182*	219	178
hsa-miR-183	266	228
hsa-miR-183	258	214
hsa-miR-183	281	231
hsa-miR-183	280	234
hsa-miR-184	225	191
hsa-miR-184	229	189
hsa-miR-184	238	186
hsa-miR-184	233	185
hsa-miR-185	266	220
hsa-miR-185	280	237
hsa-miR-185	291	225
hsa-miR-185	286	241
hsa-miR-186	229	166

hsa-miR-186	258	172
hsa-miR-186	248	166
hsa-miR-186	232	166
hsa-miR-187	252	233
hsa-miR-187	259	229
hsa-miR-187	259	214
hsa-miR-187	263	220
hsa-miR-188	239	205
hsa-miR-188	227	188
hsa-miR-188	246	201
hsa-miR-188	254	206
hsa-miR-189	199	184
hsa-miR-189	201	181
hsa-miR-189	204	185
hsa-miR-189	211	183
hsa-miR-18a	225	173
hsa-miR-18a	204	172
hsa-miR-18a	224	171
hsa-miR-18a	241	187
hsa-miR-18a	224	166
hsa-miR-18a	206	168
hsa-miR-18a	223	169
hsa-miR-18a	219	172
hsa-miR-18a*	222	183
hsa-miR-18a*	228	183
hsa-miR-18a*	229	183

hsa-miR-18a*	228	185
hsa-miR-18b	206	175
hsa-miR-18b	205	177
hsa-miR-18b	214	178
hsa-miR-18b	224	180
hsa-miR-190	279	172
hsa-miR-190	262	169
hsa-miR-190	270	165
hsa-miR-190	265	171
hsa-miR-191	725	549
hsa-miR-191	916	642
hsa-miR-191	942	599
hsa-miR-191	740	483
hsa-miR-191*	202	168
hsa-miR-191*	202	164
hsa-miR-191*	199	167
hsa-miR-191*	201	160
hsa-miR-192	179	168
hsa-miR-192	173	165
hsa-miR-192	177	170
hsa-miR-192	193	169
hsa-miR-193a	242	349
hsa-miR-193a	247	384
hsa-miR-193a	249	334
hsa-miR-193a	228	256
hsa-miR-193b	291	225

hsa-miR-193b	269	219
hsa-miR-193b	316	239
hsa-miR-193b	364	233
hsa-miR-194	201	174
hsa-miR-194	213	174
hsa-miR-194	214	183
hsa-miR-194	206	173
hsa-miR-195	203	179
hsa-miR-195	208	192
hsa-miR-195	198	184
hsa-miR-195	221	189
hsa-miR-195	212	177
hsa-miR-195	252	220
hsa-miR-195	204	174
hsa-miR-195	238	212
hsa-miR-196a	200	166
hsa-miR-196a	209	172
hsa-miR-196a	209	165
hsa-miR-196a	209	162
hsa-miR-196b	216	165
hsa-miR-196b	221	166
hsa-miR-196b	220	164
hsa-miR-196b	204	154
hsa-miR-197	287	357
hsa-miR-197	299	344
hsa-miR-197	324	347

hsa-miR-197	305	344
hsa-miR-198	303	377
hsa-miR-198	298	368
hsa-miR-198	321	355
hsa-miR-198	349	398
hsa-miR-199a	203	186
hsa-miR-199a	199	179
hsa-miR-199a	204	182
hsa-miR-199a	201	191
hsa-miR-199a*	175	164
hsa-miR-199a*	179	161
hsa-miR-199a*	190	167
hsa-miR-199a*	186	162
hsa-miR-199b	250	184
hsa-miR-199b	294	208
hsa-miR-199b	244	171
hsa-miR-199b	242	170
hsa-miR-19a	245	171
hsa-miR-19a	241	171
hsa-miR-19a	242	177
hsa-miR-19a	240	177
hsa-miR-19b	218	168
hsa-miR-19b	218	174
hsa-miR-19b	228	172
hsa-miR-19b	221	168
hsa-miR-200a	190	171

hsa-miR-200a	200	169
hsa-miR-200a	208	172
hsa-miR-200a	206	170
hsa-miR-200a*	204	185
hsa-miR-200a*	207	176
hsa-miR-200a*	209	181
hsa-miR-200a*	211	181
hsa-miR-200b	266	196
hsa-miR-200b	265	207
hsa-miR-200b	381	238
hsa-miR-200b	373	235
hsa-miR-200c	376	276
hsa-miR-200c	421	285
hsa-miR-200c	415	270
hsa-miR-200c	333	230
hsa-miR-202	245	238
hsa-miR-202	235	220
hsa-miR-202	233	228
hsa-miR-202	224	190
hsa-miR-202*	216	168
hsa-miR-202*	217	167
hsa-miR-202*	220	167
hsa-miR-202*	215	170
hsa-miR-203	212	185
hsa-miR-203	234	192
hsa-miR-203	235	189

hsa-miR-203	227	194
hsa-miR-204	431	176
hsa-miR-204	415	173
hsa-miR-204	420	170
hsa-miR-204	415	162
hsa-miR-205	222	176
hsa-miR-205	227	176
hsa-miR-205	221	170
hsa-miR-205	223	165
hsa-miR-206	204	170
hsa-miR-206	198	168
hsa-miR-206	205	167
hsa-miR-206	195	162
hsa-miR-208	215	254
hsa-miR-208	231	247
hsa-miR-208	209	243
hsa-miR-208	210	219
hsa-miR-20a	194	169
hsa-miR-20a	195	160
hsa-miR-20a	183	173
hsa-miR-20a	212	172
hsa-miR-20a	191	172
hsa-miR-20a	205	165
hsa-miR-20a	188	175
hsa-miR-20a	201	170
hsa-miR-20b	187	160

hsa-miR-20b	180	164
hsa-miR-20b	185	166
hsa-miR-20b	187	166
hsa-miR-21	248	175
hsa-miR-21	247	173
hsa-miR-21	248	177
hsa-miR-21	244	174
hsa-miR-210	195	171
hsa-miR-210	191	172
hsa-miR-210	192	170
hsa-miR-210	199	165
hsa-miR-211	196	173
hsa-miR-211	197	164
hsa-miR-211	197	167
hsa-miR-211	204	168
hsa-miR-212	216	214
hsa-miR-212	247	212
hsa-miR-212	217	221
hsa-miR-212	223	233
hsa-miR-214	264	323
hsa-miR-214	266	317
hsa-miR-214	275	318
hsa-miR-214	288	324
hsa-miR-215	184	164
hsa-miR-215	201	175
hsa-miR-215	191	164

hsa-miR-215	199	170
hsa-miR-216	177	163
hsa-miR-216	182	169
hsa-miR-216	178	163
hsa-miR-216	176	168
hsa-miR-217	201	167
hsa-miR-217	196	166
hsa-miR-217	203	164
hsa-miR-217	204	163
hsa-miR-218	217	168
hsa-miR-218	221	167
hsa-miR-218	226	174
hsa-miR-218	228	164
hsa-miR-219	177	167
hsa-miR-219	181	166
hsa-miR-219	199	167
hsa-miR-219	186	167
hsa-miR-22	231	181
hsa-miR-22	241	186
hsa-miR-22	248	184
hsa-miR-22	259	183
hsa-miR-220	270	175
hsa-miR-220	262	175
hsa-miR-220	259	170
hsa-miR-220	250	177
hsa-miR-221	245	163

hsa-miR-221	253	175
hsa-miR-221	252	177
hsa-miR-221	253	166
hsa-miR-222	222	179
hsa-miR-222	219	173
hsa-miR-222	219	175
hsa-miR-222	228	178
hsa-miR-223	240	245
hsa-miR-223	247	237
hsa-miR-223	257	247
hsa-miR-223	256	245
hsa-miR-224	184	166
hsa-miR-224	181	167
hsa-miR-224	184	166
hsa-miR-224	179	169
hsa-miR-23a	227	186
hsa-miR-23a	237	189
hsa-miR-23a	241	186
hsa-miR-23a	239	185
hsa-miR-23b	189	173
hsa-miR-23b	184	178
hsa-miR-23b	187	188
hsa-miR-23b	192	168
hsa-miR-24	241	204
hsa-miR-24	258	217
hsa-miR-24	269	213

hsa-miR-24	269	206
hsa-miR-25	211	174
hsa-miR-25	213	178
hsa-miR-25	223	185
hsa-miR-25	213	176
hsa-miR-26a	275	239
hsa-miR-26a	283	237
hsa-miR-26a	283	233
hsa-miR-26a	289	227
hsa-miR-26b	252	162
hsa-miR-26b	232	170
hsa-miR-26b	272	170
hsa-miR-26b	245	165
hsa-miR-27a	222	174
hsa-miR-27a	226	183
hsa-miR-27a	230	172
hsa-miR-27a	221	171
hsa-miR-27b	191	168
hsa-miR-27b	185	163
hsa-miR-27b	266	166
hsa-miR-27b	215	173
hsa-miR-28	175	166
hsa-miR-28	179	164
hsa-miR-28	174	163
hsa-miR-28	178	160
hsa-miR-296	644	1693

hsa-miR-296	706	1605
hsa-miR-296	588	1497
hsa-miR-296	421	1395
hsa-miR-299-3p	205	180
hsa-miR-299-3p	195	181
hsa-miR-299-3p	199	175
hsa-miR-299-3p	202	178
hsa-miR-299-5p	207	185
hsa-miR-299-5p	202	195
hsa-miR-299-5p	210	187
hsa-miR-299-5p	224	192
hsa-miR-29a	200	219
hsa-miR-29a	223	252
hsa-miR-29a	242	264
hsa-miR-29a	218	223
hsa-miR-29b	183	178
hsa-miR-29b	181	174
hsa-miR-29b	179	171
hsa-miR-29b	184	173
hsa-miR-29c	214	199
hsa-miR-29c	205	188
hsa-miR-29c	224	191
hsa-miR-29c	205	184
hsa-miR-301	187	171
hsa-miR-301	181	174
hsa-miR-301	189	165

hsa-miR-301	186	167
hsa-miR-302a	189	163
hsa-miR-302a	190	166
hsa-miR-302a	194	162
hsa-miR-302a	185	162
hsa-miR-302a*	195	164
hsa-miR-302a*	192	174
hsa-miR-302a*	199	175
hsa-miR-302a*	197	168
hsa-miR-302b	177	164
hsa-miR-302b	172	165
hsa-miR-302b	183	164
hsa-miR-302b	179	163
hsa-miR-302b*	183	165
hsa-miR-302b*	187	165
hsa-miR-302b*	187	164
hsa-miR-302b*	185	167
hsa-miR-302c	179	166
hsa-miR-302c	187	171
hsa-miR-302c	188	158
hsa-miR-302c	182	164
hsa-miR-302c*	206	179
hsa-miR-302c*	208	173
hsa-miR-302c*	204	175
hsa-miR-302c*	210	175
hsa-miR-302d	200	165

hsa-miR-302d	201	175
hsa-miR-302d	191	165
hsa-miR-302d	193	163
hsa-miR-30a-3p	175	165
hsa-miR-30a-3p	188	169
hsa-miR-30a-3p	181	167
hsa-miR-30a-3p	175	168
hsa-miR-30a-5p	190	175
hsa-miR-30a-5p	191	176
hsa-miR-30a-5p	184	171
hsa-miR-30a-5p	206	174
hsa-miR-30b	202	171
hsa-miR-30b	201	170
hsa-miR-30b	207	172
hsa-miR-30b	218	176
hsa-miR-30c	215	187
hsa-miR-30c	225	187
hsa-miR-30c	249	207
hsa-miR-30c	268	210
hsa-miR-30d	288	356
hsa-miR-30d	295	317
hsa-miR-30d	312	368
hsa-miR-30d	307	327
hsa-miR-30e-3p	210	168
hsa-miR-30e-3p	224	171
hsa-miR-30e-3p	211	172

hsa-miR-30e-3p	222	170
hsa-miR-30e-5p	217	220
hsa-miR-30e-5p	210	209
hsa-miR-30e-5p	203	191
hsa-miR-30e-5p	216	200
hsa-miR-31	176	171
hsa-miR-31	176	171
hsa-miR-31	179	173
hsa-miR-31	184	171
hsa-miR-32	191	164
hsa-miR-32	192	168
hsa-miR-32	192	165
hsa-miR-32	199	167
hsa-miR-320	436	366
hsa-miR-320	469	345
hsa-miR-320	531	395
hsa-miR-320	577	408
hsa-miR-323	186	173
hsa-miR-323	187	179
hsa-miR-323	189	173
hsa-miR-323	188	176
hsa-miR-324-3p	529	1504
hsa-miR-324-3p	500	1439
hsa-miR-324-3p	530	1544
hsa-miR-324-3p	565	1598
hsa-miR-324-5p	209	196

hsa-miR-324-5p	208	188
hsa-miR-324-5p	211	179
hsa-miR-324-5p	216	190
hsa-miR-325	188	170
hsa-miR-325	190	179
hsa-miR-325	201	176
hsa-miR-325	187	169
hsa-miR-326	330	348
hsa-miR-326	335	329
hsa-miR-326	365	341
hsa-miR-326	358	346
hsa-miR-328	208	217
hsa-miR-328	219	214
hsa-miR-328	223	231
hsa-miR-328	222	239
hsa-miR-329	186	174
hsa-miR-329	186	166
hsa-miR-329	192	166
hsa-miR-329	205	175
hsa-miR-33	201	174
hsa-miR-33	199	169
hsa-miR-33	209	169
hsa-miR-33	199	160
hsa-miR-330	193	179
hsa-miR-330	188	177
hsa-miR-330	190	177

hsa-miR-330	187	177
hsa-miR-331	271	275
hsa-miR-331	276	256
hsa-miR-331	285	265
hsa-miR-331	283	261
hsa-miR-335	212	173
hsa-miR-335	212	175
hsa-miR-335	217	168
hsa-miR-335	226	171
hsa-miR-337	172	162
hsa-miR-337	177	167
hsa-miR-337	171	164
hsa-miR-337	175	171
hsa-miR-338	194	171
hsa-miR-338	197	165
hsa-miR-338	194	163
hsa-miR-338	200	163
hsa-miR-339	223	171
hsa-miR-339	224	173
hsa-miR-339	221	172
hsa-miR-339	220	180
hsa-miR-340	193	167
hsa-miR-340	194	167
hsa-miR-340	203	171
hsa-miR-340	198	170
hsa-miR-342	216	186

hsa-miR-342	204	188
hsa-miR-342	205	179
hsa-miR-342	214	180
hsa-miR-345	231	188
hsa-miR-345	236	196
hsa-miR-345	242	198
hsa-miR-345	233	187
hsa-miR-346	314	348
hsa-miR-346	317	341
hsa-miR-346	333	347
hsa-miR-346	336	319
hsa-miR-34a	206	170
hsa-miR-34a	207	170
hsa-miR-34a	211	174
hsa-miR-34a	205	165
hsa-miR-34b	184	162
hsa-miR-34b	188	161
hsa-miR-34b	182	166
hsa-miR-34b	188	158
hsa-miR-34c	180	167
hsa-miR-34c	186	170
hsa-miR-34c	185	167
hsa-miR-34c	180	165
hsa-miR-361	279	363
hsa-miR-361	211	200
hsa-miR-361	294	333

hsa-miR-361	218	206
hsa-miR-361	297	343
hsa-miR-361	221	218
hsa-miR-361	305	332
hsa-miR-361	219	210
hsa-miR-362	187	164
hsa-miR-362	177	164
hsa-miR-362	195	173
hsa-miR-362	176	167
hsa-miR-362	191	166
hsa-miR-362	178	162
hsa-miR-362	188	163
hsa-miR-362	176	166
hsa-miR-363	194	169
hsa-miR-363	186	166
hsa-miR-363	189	166
hsa-miR-363	200	164
hsa-miR-363*	5647	2803
hsa-miR-363*	5953	2738
hsa-miR-363*	6360	2585
hsa-miR-363*	6806	2807
hsa-miR-365	293	256
hsa-miR-365	297	253
hsa-miR-365	309	265
hsa-miR-365	294	250
hsa-miR-367	226	180

hsa-miR-367	178	174
hsa-miR-367	226	177
hsa-miR-367	177	174
hsa-miR-367	212	179
hsa-miR-367	174	168
hsa-miR-367	223	179
hsa-miR-367	179	169
hsa-miR-368	183	170
hsa-miR-368	181	163
hsa-miR-368	185	165
hsa-miR-368	180	174
hsa-miR-369-3p	198	170
hsa-miR-369-3p	194	165
hsa-miR-369-3p	201	166
hsa-miR-369-3p	196	164
hsa-miR-369-5p	173	178
hsa-miR-369-5p	189	167
hsa-miR-369-5p	172	174
hsa-miR-369-5p	187	174
hsa-miR-369-5p	179	176
hsa-miR-369-5p	192	162
hsa-miR-369-5p	183	172
hsa-miR-369-5p	192	166
hsa-miR-370	306	355
hsa-miR-370	315	361
hsa-miR-370	323	342

hsa-miR-370	355	382
hsa-miR-371	197	176
hsa-miR-371	203	171
hsa-miR-371	194	173
hsa-miR-371	203	174
hsa-miR-372	233	172
hsa-miR-372	232	169
hsa-miR-372	236	168
hsa-miR-372	246	165
hsa-miR-373	184	171
hsa-miR-373	198	169
hsa-miR-373	194	169
hsa-miR-373	188	163
hsa-miR-373*	340	514
hsa-miR-373*	364	511
hsa-miR-373*	361	438
hsa-miR-373*	346	428
hsa-miR-374	195	171
hsa-miR-374	201	169
hsa-miR-374	194	172
hsa-miR-374	199	164
hsa-miR-375	253	254
hsa-miR-375	252	262
hsa-miR-375	259	249
hsa-miR-375	260	234
hsa-miR-376a	188	172

hsa-miR-376a	191	167
hsa-miR-376a	188	167
hsa-miR-376a	190	169
hsa-miR-376a*	179	170
hsa-miR-376a*	178	180
hsa-miR-376a*	178	175
hsa-miR-376a*	180	168
hsa-miR-376b	190	158
hsa-miR-376b	190	168
hsa-miR-376b	208	171
hsa-miR-376b	232	178
hsa-miR-377	212	180
hsa-miR-377	221	179
hsa-miR-377	211	172
hsa-miR-377	209	177
hsa-miR-378	198	179
hsa-miR-378	188	178
hsa-miR-378	188	173
hsa-miR-378	197	177
hsa-miR-379	213	168
hsa-miR-379	222	166
hsa-miR-379	206	166
hsa-miR-379	220	169
hsa-miR-380-3p	188	173
hsa-miR-380-3p	180	163
hsa-miR-380-3p	189	176

hsa-miR-380-3p	181	167
hsa-miR-380-5p	172	169
hsa-miR-380-5p	178	163
hsa-miR-380-5p	174	168
hsa-miR-380-5p	181	167
hsa-miR-381	232	232
hsa-miR-381	307	393
hsa-miR-381	232	215
hsa-miR-381	329	387
hsa-miR-381	235	227
hsa-miR-381	327	386
hsa-miR-381	227	228
hsa-miR-381	302	322
hsa-miR-382	199	228
hsa-miR-382	193	225
hsa-miR-382	200	224
hsa-miR-382	204	210
hsa-miR-383	181	160
hsa-miR-383	180	164
hsa-miR-383	183	163
hsa-miR-383	185	164
hsa-miR-384	192	160
hsa-miR-384	217	173
hsa-miR-384	201	163
hsa-miR-384	199	162
hsa-miR-409-3p	184	186

hsa-miR-409-3p	192	165
hsa-miR-409-3p	183	166
hsa-miR-409-3p	182	187
hsa-miR-409-3p	195	172
hsa-miR-409-3p	182	170
hsa-miR-409-3p	194	188
hsa-miR-409-3p	191	163
hsa-miR-409-3p	203	176
hsa-miR-409-3p	194	183
hsa-miR-409-3p	194	172
hsa-miR-409-3p	183	165
hsa-miR-409-5p	275	425
hsa-miR-409-5p	308	334
hsa-miR-409-5p	280	422
hsa-miR-409-5p	300	353
hsa-miR-409-5p	295	402
hsa-miR-409-5p	311	320
hsa-miR-409-5p	292	353
hsa-miR-409-5p	313	330
hsa-miR-410	190	170
hsa-miR-410	183	164
hsa-miR-410	184	165
hsa-miR-410	189	166
hsa-miR-412	191	168
hsa-miR-412	197	171
hsa-miR-412	217	176

hsa-miR-412	195	179
hsa-miR-422a	196	184
hsa-miR-422a	197	180
hsa-miR-422a	198	180
hsa-miR-422a	197	176
hsa-miR-422b	195	171
hsa-miR-422b	229	288
hsa-miR-422b	200	171
hsa-miR-422b	224	294
hsa-miR-422b	202	167
hsa-miR-422b	237	287
hsa-miR-422b	199	170
hsa-miR-422b	222	250
hsa-miR-423	347	341
hsa-miR-423	341	360
hsa-miR-423	331	353
hsa-miR-423	367	363
hsa-miR-424	192	163
hsa-miR-424	185	167
hsa-miR-424	185	167
hsa-miR-424	192	165
hsa-miR-425-3p	199	188
hsa-miR-425-3p	194	192
hsa-miR-425-3p	207	188
hsa-miR-425-3p	199	187
hsa-miR-429	190	167

hsa-miR-429	185	180
hsa-miR-429	196	175
hsa-miR-429	192	171
hsa-miR-431	182	177
hsa-miR-431	184	178
hsa-miR-431	186	170
hsa-miR-431	189	170
hsa-miR-432	196	163
hsa-miR-432	203	161
hsa-miR-432	195	162
hsa-miR-432	200	167
hsa-miR-432*	230	278
hsa-miR-432*	243	283
hsa-miR-432*	265	304
hsa-miR-432*	254	282
hsa-miR-433	208	197
hsa-miR-433	224	203
hsa-miR-433	239	197
hsa-miR-433	225	194
hsa-miR-448	184	171
hsa-miR-448	192	176
hsa-miR-448	181	171
hsa-miR-448	184	167
hsa-miR-449	171	160
hsa-miR-449	173	170
hsa-miR-449	176	166

hsa-miR-449	174	165
hsa-miR-450	369	171
hsa-miR-450	353	170
hsa-miR-450	351	171
hsa-miR-450	349	166
hsa-miR-451	198	165
hsa-miR-451	203	176
hsa-miR-451	191	168
hsa-miR-451	192	169
hsa-miR-451	197	164
hsa-miR-451	194	174
hsa-miR-451	203	169
hsa-miR-451	201	171
hsa-miR-452	291	334
hsa-miR-452	289	327
hsa-miR-452	306	320
hsa-miR-452	331	348
hsa-miR-452*	237	249
hsa-miR-452*	237	229
hsa-miR-452*	226	243
hsa-miR-452*	240	229
hsa-miR-453	209	178
hsa-miR-453	206	177
hsa-miR-453	206	177
hsa-miR-453	208	174
hsa-miR-455	206	162

hsa-miR-455	207	161
hsa-miR-455	216	166
hsa-miR-455	218	168
hsa-miR-483	344	243
hsa-miR-483	351	240
hsa-miR-483	355	248
hsa-miR-483	367	242
hsa-miR-484	413	573
hsa-miR-484	429	551
hsa-miR-484	456	519
hsa-miR-484	458	543
hsa-miR-485-3p	258	304
hsa-miR-485-3p	259	299
hsa-miR-485-3p	256	289
hsa-miR-485-3p	255	275
hsa-miR-485-5p	190	171
hsa-miR-485-5p	192	172
hsa-miR-485-5p	188	165
hsa-miR-485-5p	196	159
hsa-miR-486	272	325
hsa-miR-486	276	287
hsa-miR-486	288	282
hsa-miR-486	294	305
hsa-miR-487a	201	185
hsa-miR-487a	222	177
hsa-miR-487a	196	175

hsa-miR-487a	213	180
hsa-miR-487b	222	319
hsa-miR-487b	199	186
hsa-miR-487b	225	303
hsa-miR-487b	200	187
hsa-miR-487b	236	301
hsa-miR-487b	198	180
hsa-miR-487b	231	317
hsa-miR-487b	194	183
hsa-miR-488	194	173
hsa-miR-488	193	173
hsa-miR-488	197	173
hsa-miR-488	199	168
hsa-miR-489	248	223
hsa-miR-489	252	219
hsa-miR-489	248	221
hsa-miR-489	244	210
hsa-miR-490	220	220
hsa-miR-490	221	221
hsa-miR-490	225	218
hsa-miR-490	226	226
hsa-miR-491	205	174
hsa-miR-491	205	186
hsa-miR-491	202	171
hsa-miR-491	211	174
hsa-miR-492	350	422

hsa-miR-492	359	389
hsa-miR-492	354	375
hsa-miR-492	356	382
hsa-miR-493-3p	186	172
hsa-miR-493-3p	184	174
hsa-miR-493-3p	190	174
hsa-miR-493-3p	188	174
hsa-miR-493-5p	233	167
hsa-miR-493-5p	230	167
hsa-miR-493-5p	230	166
hsa-miR-493-5p	217	176
hsa-miR-494	389	772
hsa-miR-494	341	537
hsa-miR-494	396	841
hsa-miR-494	343	512
hsa-miR-494	407	820
hsa-miR-494	355	509
hsa-miR-494	437	896
hsa-miR-494	374	497
hsa-miR-495	203	163
hsa-miR-495	208	157
hsa-miR-495	207	164
hsa-miR-495	208	162
hsa-miR-496	219	166
hsa-miR-496	240	175
hsa-miR-496	268	171

hsa-miR-496	222	164
hsa-miR-497	203	167
hsa-miR-497	208	173
hsa-miR-497	202	169
hsa-miR-497	205	171
hsa-miR-498	361	509
hsa-miR-498	362	489
hsa-miR-498	385	513
hsa-miR-498	380	474
hsa-miR-499	179	169
hsa-miR-499	552	168
hsa-miR-499	182	171
hsa-miR-499	342	163
hsa-miR-499	185	170
hsa-miR-499	334	172
hsa-miR-499	181	170
hsa-miR-499	348	168
hsa-miR-500	472	455
hsa-miR-500	483	431
hsa-miR-500	425	359
hsa-miR-500	512	437
hsa-miR-501	230	176
hsa-miR-501	221	179
hsa-miR-501	227	182
hsa-miR-501	236	187
hsa-miR-502	210	168

hsa-miR-502	211	169
hsa-miR-502	206	170
hsa-miR-502	211	174
hsa-miR-503	315	385
hsa-miR-503	318	358
hsa-miR-503	322	361
hsa-miR-503	321	373
hsa-miR-504	190	170
hsa-miR-504	226	179
hsa-miR-504	191	171
hsa-miR-504	194	168
hsa-miR-505	186	171
hsa-miR-505	182	170
hsa-miR-505	185	170
hsa-miR-505	183	174
hsa-miR-505	189	162
hsa-miR-505	181	167
hsa-miR-505	186	167
hsa-miR-505	179	168
hsa-miR-506	204	206
hsa-miR-506	208	196
hsa-miR-506	213	194
hsa-miR-506	208	195
hsa-miR-507	208	166
hsa-miR-507	215	167
hsa-miR-507	230	167

hsa-miR-507	212	162
hsa-miR-508	195	162
hsa-miR-508	223	169
hsa-miR-508	207	163
hsa-miR-508	196	161
hsa-miR-509	186	161
hsa-miR-509	183	164
hsa-miR-509	183	161
hsa-miR-509	197	173
hsa-miR-510	245	282
hsa-miR-510	243	283
hsa-miR-510	247	280
hsa-miR-510	273	268
hsa-miR-511	188	161
hsa-miR-511	187	165
hsa-miR-511	187	167
hsa-miR-511	188	169
hsa-miR-512-3p	189	169
hsa-miR-512-3p	203	172
hsa-miR-512-3p	200	169
hsa-miR-512-3p	191	165
hsa-miR-512-5p	342	373
hsa-miR-512-5p	314	311
hsa-miR-512-5p	366	348
hsa-miR-512-5p	360	336
hsa-miR-513	349	849

hsa-miR-513	404	770
hsa-miR-513	397	775
hsa-miR-513	412	724
hsa-miR-514	211	164
hsa-miR-514	211	160
hsa-miR-514	202	162
hsa-miR-514	214	162
hsa-miR-515-3p	192	174
hsa-miR-515-3p	220	183
hsa-miR-515-3p	201	176
hsa-miR-515-3p	198	176
hsa-miR-515-5p	192	176
hsa-miR-515-5p	209	175
hsa-miR-515-5p	192	169
hsa-miR-515-5p	193	170
hsa-miR-516-3p	195	179
hsa-miR-516-3p	205	178
hsa-miR-516-3p	207	183
hsa-miR-516-3p	205	185
hsa-miR-516-5p	205	187
hsa-miR-516-5p	204	181
hsa-miR-516-5p	204	178
hsa-miR-516-5p	205	182
hsa-miR-517*	193	175
hsa-miR-517*	199	166
hsa-miR-517*	192	168

hsa-miR-517*	210	173
hsa-miR-517a	193	163
hsa-miR-517a	215	184
hsa-miR-517a	208	177
hsa-miR-517a	203	177
hsa-miR-517a-517b	195	174
hsa-miR-517a-517b	202	176
hsa-miR-517a-517b	198	176
hsa-miR-517a-517b	200	167
hsa-miR-517c	196	161
hsa-miR-517c	196	158
hsa-miR-517c	200	168
hsa-miR-517c	200	164
hsa-miR-518a	196	168
hsa-miR-518a	191	167
hsa-miR-518a	195	161
hsa-miR-518a	196	162
hsa-miR-518b	389	430
hsa-miR-518b	382	390
hsa-miR-518b	372	406
hsa-miR-518b	387	396
hsa-miR-518c	187	167
hsa-miR-518c	188	163
hsa-miR-518c	185	166
hsa-miR-518c	200	163
hsa-miR-518c*	527	543

hsa-miR-518c*	549	552
hsa-miR-518c*	555	537
hsa-miR-518c*	577	556
hsa-miR-518d	186	173
hsa-miR-518d	193	172
hsa-miR-518d	194	173
hsa-miR-518d	190	171
hsa-miR-518e	202	170
hsa-miR-518e	200	169
hsa-miR-518e	199	168
hsa-miR-518e	213	169
hsa-miR-518f	187	171
hsa-miR-518f	200	186
hsa-miR-518f	188	168
hsa-miR-518f	188	167
hsa-miR-518f*-526a	315	462
hsa-miR-518f*-526a	335	516
hsa-miR-518f*-526a	347	519
hsa-miR-518f*-526a	326	416
hsa-miR-519a	193	163
hsa-miR-519a	190	162
hsa-miR-519a	192	161
hsa-miR-519a	191	161
hsa-miR-519b	196	166
hsa-miR-519b	190	166
hsa-miR-519b	197	164

hsa-miR-519b	191	164
hsa-miR-519c	198	166
hsa-miR-519c	204	176
hsa-miR-519c	197	167
hsa-miR-519c	196	165
hsa-miR-519d	247	199
hsa-miR-519d	240	188
hsa-miR-519d	237	180
hsa-miR-519d	261	192
hsa-miR-519e	195	171
hsa-miR-519e	210	178
hsa-miR-519e	202	167
hsa-miR-519e	195	170
hsa-miR-519e*	280	310
hsa-miR-519e*	288	310
hsa-miR-519e*	290	318
hsa-miR-519e*	292	288
hsa-miR-520a	184	167
hsa-miR-520a	192	168
hsa-miR-520a	189	166
hsa-miR-520a	186	171
hsa-miR-520a*	205	206
hsa-miR-520a*	205	210
hsa-miR-520a*	215	204
hsa-miR-520a*	212	196
hsa-miR-520b-520c	192	172

hsa-miR-520b-520c	186	165
hsa-miR-520b-520c	194	168
hsa-miR-520b-520c	197	169
hsa-miR-520d	264	172
hsa-miR-520d	261	171
hsa-miR-520d	313	203
hsa-miR-520d	252	175
hsa-miR-520d*	239	206
hsa-miR-520d*	236	210
hsa-miR-520d*	234	207
hsa-miR-520d*	272	199
hsa-miR-520e	186	163
hsa-miR-520e	190	161
hsa-miR-520e	193	170
hsa-miR-520e	193	168
hsa-miR-520f-520c	206	173
hsa-miR-520f-520c	205	171
hsa-miR-520f-520c	200	180
hsa-miR-520f-520c	200	173
hsa-miR-520g	185	168
hsa-miR-520g	185	162
hsa-miR-520g	181	168
hsa-miR-520g	195	166
hsa-miR-520g-520h	187	168
hsa-miR-520g-520h	192	164
hsa-miR-520g-520h	186	166

hsa-miR-520g-520h	196	171
hsa-miR-521	191	169
hsa-miR-521	195	167
hsa-miR-521	182	159
hsa-miR-521	193	168
hsa-miR-522	211	165
hsa-miR-522	210	177
hsa-miR-522	209	170
hsa-miR-522	205	166
hsa-miR-523	186	170
hsa-miR-523	195	170
hsa-miR-523	187	168
hsa-miR-523	202	187
hsa-miR-524*	207	179
hsa-miR-524*	202	179
hsa-miR-524*	207	181
hsa-miR-524*	208	180
hsa-miR-525	245	225
hsa-miR-525	241	230
hsa-miR-525	252	246
hsa-miR-525	250	223
hsa-miR-525*-524	209	200
hsa-miR-525*-524	203	185
hsa-miR-525*-524	206	192
hsa-miR-525*-524	215	194
hsa-miR-526b	243	276

hsa-miR-526b	261	283
hsa-miR-526b	243	268
hsa-miR-526b	250	274
hsa-miR-526b*	249	163
hsa-miR-526b*	238	173
hsa-miR-526b*	248	174
hsa-miR-526b*	237	176
hsa-miR-526c	255	270
hsa-miR-526c	246	276
hsa-miR-526c	262	262
hsa-miR-526c	254	230
hsa-miR-527-518a-2*	313	365
hsa-miR-527-518a-2*	309	375
hsa-miR-527-518a-2*	325	388
hsa-miR-527-518a-2*	318	324
hsa-miR-539	178	165
hsa-miR-539	170	163
hsa-miR-539	173	167
hsa-miR-539	179	168
hsa-miR-542-3p	169	177
hsa-miR-542-3p	188	169
hsa-miR-542-3p	175	179
hsa-miR-542-3p	189	163
hsa-miR-542-3p	179	179
hsa-miR-542-3p	187	164
hsa-miR-542-3p	178	178

hsa-miR-542-3p	187	175
hsa-miR-542-5p	195	166
hsa-miR-542-5p	191	170
hsa-miR-542-5p	195	168
hsa-miR-542-5p	216	170
hsa-miR-544	175	158
hsa-miR-544	196	164
hsa-miR-544	186	163
hsa-miR-544	187	170
hsa-miR-545	178	169
hsa-miR-545	189	174
hsa-miR-545	184	171
hsa-miR-545	177	162
hsa-miR-7	227	169
hsa-miR-7	231	165
hsa-miR-7	229	171
hsa-miR-7	248	171
hsa-miR-7	237	168
hsa-miR-7	249	172
hsa-miR-7	232	169
hsa-miR-7	232	170
hsa-miR-9	181	163
hsa-miR-9	189	174
hsa-miR-9	187	170
hsa-miR-9	178	163
hsa-miR-9*	186	172

hsa-miR-9*	186	172
hsa-miR-9*	188	173
hsa-miR-9*	186	163
hsa-miR-92	199	172
hsa-miR-92	216	178
hsa-miR-92	200	177
hsa-miR-92	208	170
hsa-miR-93	195	165
hsa-miR-93	251	176
hsa-miR-93	203	175
hsa-miR-93	199	173
hsa-miR-95	192	183
hsa-miR-95	208	190
hsa-miR-95	187	188
hsa-miR-95	201	185
hsa-miR-96	191	164
hsa-miR-96	192	168
hsa-miR-96	196	170
hsa-miR-96	190	171
hsa-miR-98	211	185
hsa-miR-98	207	183
hsa-miR-98	215	176
hsa-miR-98	216	175
hsa-miR-99a	295	221
hsa-miR-99a	278	225
hsa-miR-99a	281	206

hsa-miR-99a	310	205
hsa-miR-99b	254	257
hsa-miR-99b	251	244
hsa-miR-99b	274	259
hsa-miR-99b	298	262

Supplementary Table 2

Analysis of concondance of changes between the differentially expressed lists of NEPC/PCa and CL1/LNCaP comparisons							
**SYMBOL**	**Description**	**NEPC/Pca Comparison Raw.pvalue**	**NEPC/Pca Change Direction**	**LNCaP/CL1 ratio**	**LNCaP (TPM*)**	**CL1 (TPM)**	**Concordance of Changes**
GTSE1	G-2 and S-phase expressed 1|Homo sapiens B99 mRNA, complete cds.	1.51E-06	UP	0	0	39	Yes
ARNTL2	aryl hydrocarbon receptor nuclear	2.25E-05	UP	0	0	62	Yes
NUP214	nucleoporin 214kDa|Homo sapiens KIAA0023 mRNA for KIAA0023 splice variant 1, partial cds.	1.47E-05	UP	0.038961038	3	77	Yes
ENO2	enolase 2	3.79E-06	UP	0.114503816	15	131	Yes
DAZAP1	DAZ associated protein 1 isoform b|DAZ associated protein 1 isoform a	7.40E-06	UP	0.18229167	35	192	Yes
EXO1	exonuclease 1 isoform b|exonuclease 1 isoform a	9.04E-09	UP	0.21276595	20	94	Yes
MCM2	minichromosome maintenance complex component 2|Homo sapiens cDNA FLJ46429 fis, clone THYMU3014372, highly similar to DNA replication licensing factor MCM2.	3.11E-05	UP	0.2264151	24	106	Yes
PIP5K1C	phosphatidylinositol-4-phosphate 5-kinase, type	1.02E-05	UP	0.23880596	16	67	Yes
RRM2	ribonucleotide reductase M2 polypeptide	1.02E-05	UP	0.26006192	252	969	Yes
CENPF	centromere protein F	1.01E-07	UP	0.26978418	75	278	Yes
CDCA4	cell division cycle associated 4 isoform 14|cell division cycle associated 4 isoform 13	6.41E-06	UP	0.31428573	44	140	Yes
RNASEH2A	ribonuclease H2, large subunit	7.05E-06	UP	0.33146068	59	178	Yes
KIF4A	kinesin family member 4	1.48E-08	UP	0.34693876	51	147	Yes
CFL1	Homo sapiens cDNA FLJ40371 fis, clone TESTI2034931, highly similar to Cofilin-1.	1.28E-06	UP	0.3558648	179	503	Yes
CFL1	cofilin 1 (non-muscle)	3.17E-06	UP	0.3558648	179	503	Yes
TOP2A	DNA topoisomerase II, alpha isozyme|Homo sapiens topoisomerase II alpha-2 (TOP2A) mRNA, partial cds.	9.09E-06	UP	0.43125	69	160	Yes
SLC25A5	solute carrier family 25, member 5	2.11E-08	UP	0.5057113	487	963	Yes
ZWINT	ZW10 interactor isoform a|ZW10 interactor isoform b|Uncharacterized	9.23E-06	UP	0.6478261	298	460	Yes

	protein ZWINT.						
RPS27L	Homo sapiens 40S ribosomal protein S27 isoform mRNA, complete cds.|ribosomal protein S27-like	1.78E-07	DOWN	2.0719697	547	264	Yes
HMG20B	high-mobility group 20B	1.11E-06	DOWN	2.2	165	75	Yes
MIPEP	mitochondrial intermediate peptidase	4.00E-06	DOWN	6.571429	92	14	Yes
CHSY1	carbohydrate (chondroitin) synthase 1	1.23E-09	DOWN	8.357143	117	14	Yes
IMMP2L	IMP2 inner mitochondrial membrane protease-like	6.44E-06	DOWN	35	35	1	Yes
GLUD1	glutamate dehydrogenase 1	1.95E-05	DOWN	55	55	1	Yes
BBS4	Bardet-Biedl syndrome 4|Homo sapiens clone HQ0692.	2.57E-05	DOWN	35999.996	36	0.001	Yes
RLN2	relaxin 2 isoform 2|relaxin 2 isoform 1 preproprotein	8.10E-08	DOWN	39999.996	40	0.001	Yes
AZGP1	alpha-2-glycoprotein 1, zinc	2.91E-05	DOWN	41999.996	42	0.001	Yes
ASAH1	N-acylsphingosine amidohydrolase 1 isoform a|N-acylsphingosine amidohydrolase 1 isoform b|N-acylsphingosine amidohydrolase 1 isoform c	2.15E-05	DO	21.333334	64	3	Yes
PPIA	peptidylprolyl isomerase A|Homo sapiens cDNA: FLJ22916 fis, clone KAT06406, highly similar to HSCYCR Human mRNA for T-cell cyclophilin.	2.99E-07	UP	1.2550478	4724	3764	No
BUB1B	BUB1 budding uninhibited by benzimidazoles 1	8.57E-06	UP	2.3253012	193	83	No
ZIC2	zinc finger protein of the cerebellum 2	2.57E-05	UP	6.3636365	70	11	No
SYT4	synaptotagmin IV|Homo sapiens mRNA for KIAA1342 protein, partial cds.|Homo sapiens cDNA, FLJ99422.	3.27E-06	UP	39999.996	40	0.001	No
NSMAF	neutral sphingomyelinase (N-SMase) activation	3.40E-08	DOWN	0	0	61	No
ANXA10	annexin A10	6.64E-07	DOWN	0	0	69	No
FGF2	fibroblast growth factor 2	2.42E-05	DOWN	0	0	47	No
*TPM, transcripts per million							

Supplementary Table 3

Linear association between XRN1 and miR-204 expression

	N	coefficients	p value
**Total samples**	171	-0.134	0.080
**BPH**	45	-0.304	0.043
**PCa**	126	-0.078	0.387
**Recurrence**			
**no**	50	-0.155	0.282
**yes**	70	-0.083	0.494
**Gleason scores**			
**<7**	44	0.114	0.450
**≥7**	74	-0.269	0.021
**nonrecurrence**	20	-0.533	0.028
**recurrence**	54	-0.154	0.257
**Pathological stage**			
**pT2–pT3a**	101	-0.104	0.302
**pT3b**	18	-0.124	0.624

Supplemetary table 4

List of the RT-PCR primer sequences used in the present study

Primers	Sequence (from 5' to 3')
RT-miR-204(human)	GTCGTATCCAGTGCGTGTCGTGGAGTCGGCAATTGCACTGGATACGACAGGCATA
RT- miR-204 (rat)	GTCGTATCCAGTGCGTGTCGTGGAGTCGGCAATTGCACTGGATACGACAGGCATA
RT-U6 (human)	CGCTTCACGAATTTGCGTGTCAT
RT-U6 (rat)	CGCTTCACGAATTTGCGTGTCAT
miR-204 (human)-F	GGTTCCCTTTGTCATCC
miR-204 (human)-R	TGCGTGTCGTGGAGTC
miR-204 (rat)-F	GGTTCCCTTTGTCATCC
miR-204 (rat)-R	TGCGTGTCGTGGAGTC
U6 (human)-F	CTTCGGCAGCACATATACTAAAAT
U6 (human)-R	CGCTTCACGAATTTGCGTGTCAT
U6 (rat)-F	GCTTCGGCAGCACATATACTAAAAT
U6 (rat)-R	CGCTTCACGAATTTGCGTGTCAT
XRN1(human)-F	GGAAACAACAGGAATGGGAAGC
XRN1(human)-R	ACCAGCACATTAGGCACTCAC
XRN1(rat)-F	GGAAACAACAGGAATGGGAAGC
XRN1(rat)-R	ACCAGCACATTAGGCACTCAC
CD44-F	AGCAACCAAGAGGCAAGAAA
CD44-R	GTGTGGTTGAAATGGTGCTG
GAPDH (human)-F	GGTGAAGGTCGGAGTCAACGGA
GAPDH (human)-R	GAGGGATCTCGCTCCTGGAAGA
β-actin (rat)-F	CTGTCCCTGTATGCCTCTGGTC
β-actin (rat)-R	TGAGGTAGTCCGTCAGGTCCC
